# Assessing life-space mobility: A systematic review of questionnaires and their psychometric properties

**DOI:** 10.1007/s00391-022-02035-5

**Published:** 2022-03-04

**Authors:** Phoebe Ullrich, Christian Werner, Bastian Abel, Merit Hummel, Jürgen M. Bauer, Klaus Hauer

**Affiliations:** Geriatrisches Zentrum am Universitätsklinikum Heidelberg, AGAPLESION Bethanien Krankenhaus, Rohrbacher Str. 149, 69126 Heidelberg, Germany

**Keywords:** Diagnostic self-evaluation, Validation, Environment/spatial area, Aged, Mobility limitation, Diagnostische Selbsteinschätzung, Validierung, Umwelt/räumliche Umgebung, Ältere Menschen, Mobilitätseinschränkung

## Abstract

**Background:**

Life-space mobility (LSM), as the extent of mobility within one’s environment, is a key for successful aging and has become a relevant concept in gerontology and geriatric research. Adequate assessment instruments are needed to identify older persons with LSM restrictions, and to initiate, adapt or evaluate intervention strategies.

**Objective:**

To systematically identify, describe and analyze the psychometric properties of LSM questionnaires, with a special focus on their availability in the German language.

**Methods:**

A systematic literature search was conducted in PubMed, PsycINFO, Cochrane Library, CINAHL, and Web of Science. Studies that examined at least one psychometric property of LSM questionnaires published up to August 2021 were included and evaluated based on the consensus-based standards for the selection of health measurement instruments (COSMIN) guidelines.

**Results:**

This study included 37 validation studies describing 13 different LSM questionnaires. Methodological quality and comprehensiveness of validations were heterogeneous. Based on comprehensive and high-quality results, four LSM questionnaires stood out: the University of Alabama at Birmingham life-space assessment (UAB-LSA), life-space assessment in persons with cognitive impairment (LSA-CI), interview-based and proxy-based versions of the life-space assessment in institutionalized settings (LSA-IS), all of them available in the German language.

**Conclusion:**

This systematic review provides a concise overview of available LSM questionnaires and their psychometric properties to facilitate the selection for use in clinical practice and research. The UAB-LSA and LSA-CI for community settings and the interview-based or proxy-based LSA-IS for institutional settings were found to be the most appropriate LSM questionnaires.

**Supplementary Information:**

The online version of this article (10.1007/s00391-022-02035-5) contains supplementary material, which is available to authorized users.

## Abstract (de)

**Hintergrund:**

Life-space-Mobilität (LSM) als das Ausmaß der Mobilität in der Umgebung ist ein Schlüssel für erfolgreiches Altern und hat sich zu einem relevanten Konzept in der Gerontologie und Geriatrie entwickelt. Geeignete Assessment-Instrumente werden benötigt, um ältere Personen mit Einschränkungen in der LSM zu identifizieren und Interventionen einzuleiten, anzupassen oder zu evaluieren.

**Ziel:**

Systematische Identifikation, Beschreibung und Analyse psychometrischer Eigenschaften von LSM-Fragebögen und deren Verfügbarkeit in deutscher Sprache.

**Methoden:**

Eine systematische Literatursuche in PubMed, PsyCINFO, Cochrane Library, CINAHL und Web of Science wurde durchgeführt. Bis August 2021 publizierte Studien zu psychometrischen Eigenschaften von LSM-Fragebögen wurden eingeschlossen und basierend auf den „Consensus-based standards for the selection of health measurement instruments“(COSMIN)-Richtlinien bewertet.

**Ergebnisse:**

Diese Studie schloss 37 Validierungsstudien für 13 verschiedene LSM-Fragebögen ein. Die methodische Qualität und der Umfang der Validierungen waren heterogen. Basierend auf umfassenden und qualitativ hochwertigen Ergebnissen sind 4 Fragebögen hervorzuheben: „University of Alabama at Birmingham life-space assessment“ (UAB-LSA), „Life-space assessment in persons with cognitive impairment“ (LSA-CI), interview- und proxybasierte Versionen des „Life-space assessment in institutionalized settings“ (LSA-IS), die alle in Deutsch verfügbar sind.

**Diskussion:**

Dieses systematische Review gibt einen kompakten Überblick über LSM-Fragebögen und deren psychometrische Eigenschaften. Als LSM-Fragebögen sind der UAB-LSA und der LSA-CI für kommunale Settings und der interview- oder proxybasierte LSA-IS für institutionelle Settings am besten geeignet.

## Introduction

Mobility is a key factor for successful aging. In clinical practice and research, assessing mobility is important for identifying individuals at risk or with mobility limitations, developing and adapting intervention strategies, and evaluating intervention effectiveness. Various mobility instruments have been developed, with a focus on assessing motor capacity [1]; however, capacity and habitual performance are different concepts [2]. Assessment instruments on life-space mobility (LSM), defined as the spatial extent of movement in daily life, represents an extension of capacity-oriented mobility instruments by a behavioral perspective of habitual mobility performance in everyday life [3, 4].

Despite technical developments such as the global positioning system (GPS) to objectively measure life-space parameters [5], LSM has so far predominantly been assessed via questionnaires, allowing an easy to implement, low resource, highly accepted and valid assessment of combined indoor and outdoor mobility, and contextual aspects of mobility (e.g., mobility aids, personal assistance).

Selecting an appropriate questionnaire for clinical practice and research is challenging. Setting and target population as well as practical aspects (e.g., interview duration, equipment required), and the methodological quality of the instrument determined by psychometric properties (such as feasibility, validity, reliability, and sensitivity to change) must be considered. The use of instruments validated in the appropriate language and cultural setting represents a mandatory methodological criterion, which has so far not been documented for LSM questionnaires in the German language. In addition, previous reviews of LSM measurement tools focused only on specific LSM questionnaires [6] or did not provide detailed information on their psychometric properties and did not include new instruments developed in the past years [3].

Thus, the primary aim of the present systematic review was to provide an overview on currently available LSM questionnaires and their psychometric properties to facilitate the selection for use in clinical practice and research. A secondary aim was to identify LSM questionnaires validated for use in the German language.

## Methods

A systematic literature search was conducted in accordance with recommendations from the Preferred Reporting Items for Systematic review and Meta-Analysis (PRISMA) protocols [7] and the consensus-based standards for the selection of health measurement instruments (COSMIN) guidelines for systematic reviews of patient-reported outcome measures [8].

We performed a complete database search with no language restrictions in CINAHL, Cochrane Library, PsycInfo, Pubmed, and Web of Science up to August 2021. Search terms included combinations of variants for the keyword life-space along with different terms for assessment, validation, and psychometric properties (see Table S1), as recommended elsewhere [9].

Titles, abstracts and full texts of studies identified by the search were screened for eligibility by two independent reviewers (PU, BA). Any disagreements were solved by a third reviewer (KH). Reference lists of relevant articles and reviews, and grey literature were also screened for additional studies for inclusion.

Studies eligible for inclusion provided empirical evidence for validity, reliability, sensitivity to change, and/or feasibility of a questionnaire designed to assess more than two spatial aspects of mobility (e.g. not only indoor vs. outdoor), usually described as life-space mobility (LSM). Each version of a LSM questionnaire with relevant changes to the assessment procedure or the information collected were considered as a separate instrument.

Data extraction was performed by two independent reviewers (PU, BA). Information on main features of the identified questionnaires (i.e., mobility construct, target population, test administration, data collection, and scoring) was extracted from the original article of each questionnaire. Information on different aspects of feasibility, validity, reliability, and sensitivity to change was gathered from all included studies. The methodological quality of the included studies was rated by three reviewers (PU, BA, MH) using the COSMIN risk of bias checklist, considering additional references for sample size rating [10, 11]. Disagreements in ratings were resolved by consensus. Where applicable, results on the same questionnaire for the main domains of psychometric properties (feasibility, validity, reliability, sensitivity to change) were summarized from multiple studies (i.e., studies in different populations or different language versions of the same questionnaire) to give an overall recommendation for use. To make the quality rating transparent, notes on the methodological limitations of the studies in the evaluation of the psychometric properties were also provided in the data extraction.

An additional focus of the narrative analysis was lain on the availability of LSM questionnaires validated in the German language.

## Results

**Fig. 1:**
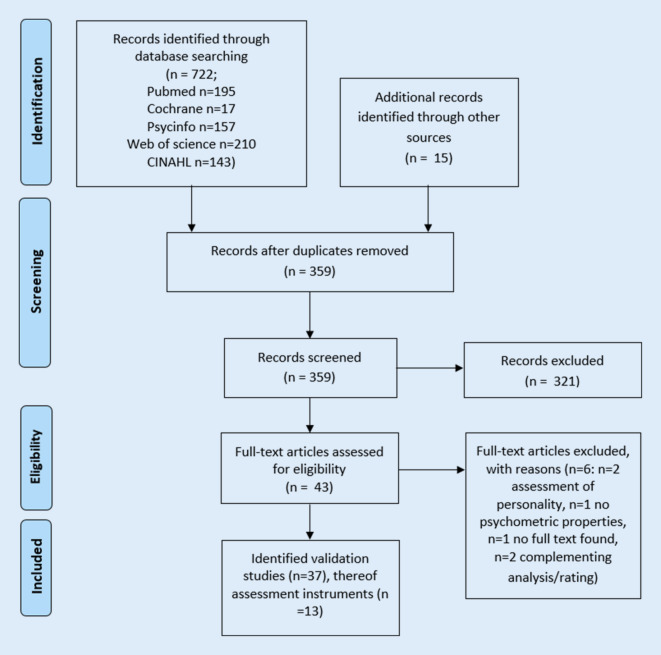
Flowchart for search process

The literature search yielded 37 studies eligible for inclusion (see Fig. 1), in which 13 unique LSM questionnaires were identified: life-space diary (LSD) [12], nursing home life-space diameter (NHLSD) [13], life-space questionnaire (LSQ) [14], University of Alabama at Birmingham-life-space assessment (UAB-LSA) [15], phone-based (LSA-F) [16] and proxy-based LSA (LSA companion) [17], homebound mobility assessment (HBMA) [18], indoor life-space mobility at home (LSH) [19], home-based life-space assessment (Hb-LSA) [20], life-space assessment for persons with cognitive impairment (LSA-CI) [21], interview-based [22] and proxy-reported life-space assessment for institutionalized settings (LSA-IS) [23], and map-based life-space assessment (MBA) [24].

### Characteristics of the LSM questionnaires

A detailed description of these questionnaires is provided in Table S2. The majority of them consider not only spatial (e.g., distance) but also contextual aspects (e.g., mobility aids, personal assistance) of mobility. Nine questionnaires have initially been developed for community-dwelling older persons (LSD, LSQ, UAB-LSA, LSA‑F, LSA-CI and MBA) [12, 14–16, 18–21, 24], with three of them specifically focusing on persons having difficulties in going outdoors (HBMA, LSH and Hb-LSA) [18–20], one for power mobility device users (LSA companion) [16], and three for institutionalized older persons (NHLSD, LSA-IS self-report and proxy versions) [13, 22, 23]. The majority of questionnaires are based on self-reports by structured interviews, with one being a web-based survey (MBA) [24]. The NHLSD is the only one specifically designed for proxy documentation of nurses [13]. Four self-report questionnaires are available as proxy reports (LSA companion, LSA-IS) [17, 23] or have used proxy reports (HBMA, LSH) [18, 19]. All self-report questionnaires assume full mental abilities of the respondent, except for two (LSA-CI, LSA-IS) [21, 22]. Most questionnaires are conducted via face-to-face interview, two can also be performed via phone (HBMA, LSA-F) [16, 18]. Specific equipment is only needed for the web-based MBA, while all other questionnaires were designed as paper and pencil tests. The observation period ranges between 24 h [22, 23] and 4 weeks or 1 month [12, 15–17, 20]. Rating for the different questionnaires varied with total scores ranging from 0–8 points [18] to 0–120 points [15–17, 22, 23], with 2 questionnaires having an open-ended score (LSH, MBA) [19, 24]. Higher scores consistently indicate higher mobility levels. Some questionnaires provide options to analyze only spatial mobility aspects [15, 16, 21–23], rate specific sub-groups depending on frequency of being outdoors [12] or differentiate between low or high [25], or low, intermediate, and high LSM [18].

### Psychometric properties

**Fig. 2:**
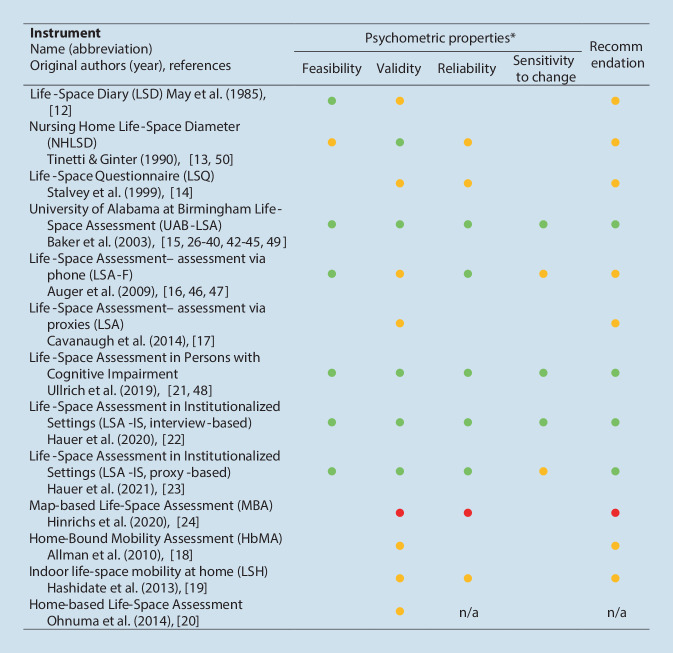
Overview on included assessment instruments and their psychometric properties. *Asterisk* Rating considers quality and results, *green* good, *yellow* moderate, *red* low, *n/a* data not available

Figure 2 summarizes the results on the psychometric properties and recommendations for each questionnaire based on the 37 included validation studies. Most studies (*n* = 19) were identified for evaluating the psychometric properties of UAB-LSA [15] in different languages or populations [26–45], additionally the phone-based [16, 46, 47] and proxy-reported questionnaires [17] are also based on the UAB-LSA. Detailed information on the individual studies, questionnaires, and COSMIN quality ratings is provided in Table S3.

Sample sizes of the validation studies ranged from 5 [16] to 2147 [32] participants, with 16 studies having less than 50 participants. Mean age of participants was 75 years, ranging from 43 years to 85 years. Studies included community-dwelling older persons without [12, 14, 15, 17–20, 24, 26–28, 31–36, 38, 39, 47, 48] and with cognitive impairment [21], persons with power mobility devices [16], persons with chronic obstructive pulmonary disease [43–45], persons with stroke [40, 42], persons with vestibular disorders [37], patients with Parkinson disease [49], persons with spinal cord injury [46], persons with critical illness [29], persons in palliative care [30], geriatric inpatients [22, 23, 50], and nursing home residents [13]. Construct and concurrent validity, and test-retest reliability were the most frequently evaluated psychometric properties.

### Feasibility

Floor or ceiling effects were analyzed in 16 studies, with all reporting absence of such effects [12, 13, 15, 16, 20–23, 31, 36, 42–44, 46–48, 50]. Completion rates ranged from 82.6% to 100% but were only rarely reported [12, 16, 21–23, 47, 48]. Completion times were provided even less frequently, averaging between 3 min and 9 min across 5 studies [16, 21, 22, 27, 48], with phone-based questionnaires taking longer.

### Validity

Almost all studies included a validity analysis. Construct validity was assessed in 30 studies [12–15, 18–23, 26, 27, 29, 30, 32–38, 40, 42–48, 50], using 1–20 construct variables of different domains (e.g., physical, cognitive, psychosocial, financial, environmental, and/or sociodemographic status). Four studies used a conceptual framework for mobility [4] to select construct variables [21–23, 48]. Overall, results on construct validity yielded expected directions and magnitudes of correlations with cut-offs of *r* > 0.5 for similar (e.g., physical variables) and *r* > 0.30–0.50 for related constructs (e.g., financial, psychosocial variables) for evaluation. Concurrent validity was assessed in seven studies [17, 19, 20, 23, 24, 49, 50]. Apart from two studies [19, 24], concurrent validity results ranged from acceptable to good, depending on the closeness of the comparison instrument to the concept of the LSM questionnaires. Content validity was assessed with positive results in four studies (all for the UAB-LSA) [16, 27, 28, 36], with two focusing on the translation process [16, 28].

### Reliability

Reliability results were evaluated in terms of test-retest reliability in 22 studies, showing predominantly acceptable to good results with intraclass correlation coefficients or Kappas of > 0.7 [11]. Poor test-retest reliability was reported in only one study for the MBA [24]. Acceptable interrater reliability was observed in three studies [13, 19, 27].

### Sensitivity to change

Only eight studies analyzed sensitivity to change of the LSM questionnaires [15, 21–23, 31, 35, 47, 48]. Most of them evaluated the ability to detect intervention-induced changes [21–23, 35, 47, 48]. Changes over time were analyzed in only two studies [15, 31]. For the analysis, most studies used distribution-based methods [21–23, 35, 47, 48], with one of them also using an anchor-based method [31]. Studies calculating standardized response means reported small (< 0.5) [23, 35], moderate (> 0.5) [21, 47], and large (> 0.8) [22, 48] sensitivity to intervention-induced changes.

### Risk of bias

The methodological quality of the included validation studies assessing the different psychometric properties was predominantly moderate to high across studies (Table S3). Overall, methodological quality of validity studies was predominantly negatively affected by low sample sizes that were not statistically justified [12, 17, 19, 40, 42, 50] or low number of construct variables [12, 18, 36, 43, 44, 46, 47]. Reliability analyses were limited due to very long time period between test and retest (1 year) [14], different test conditions used for test and retest (face-to-face vs. phone) [15], analysis of only relative agreement [13], and small sample sizes [13, 19, 26, 31, 34, 37, 40, 42]. For sensitivity to change, no adequate statistical analysis method was observed in one study [15], and low sample size in another [48]. Five studies could not be rated due to insufficient information as only the abstract was available [20, 29, 32, 35, 45]. A comprehensive validation approach to assess several psychometric properties with consistently high methodological quality was conducted in two studies [21, 22], and in four studies with moderate to high quality [13, 16, 23, 48]. For the UAB-LSA [15] as the most frequently evaluated LSM questionnaire, methodological quality of the studies was heterogeneous and ranged from low to high, with most of them showing, however, higher methodological quality.

### Availability in the German language

The LSA-CI and both versions of the LSA-IS were comprehensively validated in the German language, with overall good results on feasibility, validity, reliability, and sensitivity to change [21–23, 48]. A German version is also available for the UAB-LSA, whose construct validity has recently been documented in German community-dwelling older adults [33].

## Discussion

This systematic review provides a concise overview of currently available questionnaires to assess LSM and their psychometric properties with comprehensive information in the supplements. To the best of our knowledge, this is the first review to systematically analyze such a wide range of LSM questionnaires, with the aim of providing clinicians and researchers with practical knowledge on LSM questionnaires to help them select an instrument appropriate for their purpose.

A large number of different LSM questionnaires were identified. Given the importance of LSM-associated factors with aspects of successful aging (such as absence of diseases, high cognitive and physical function, active engagement with life) [6, 15] and the increased risk and prevalence of LSM limitations in older age, it was not surprising that a clear focus of these questionnaires was the development and validation for use in older people. According to the different living conditions in older age, LSM questionnaires are available for community-based and institutional settings.

Apart from appropriateness of the instrument for the target population, which also covers the availability in the specific language, its feasibility plays an important role in both clinical routine and research. Although feasibility aspects of the LSM questionnaires were rarely reported or only as side notes, presented results for the completion rates/times and floor/ceiling effects suggest that they are accepted and not time consuming and cover an adequate range of respondents’ LSM.

Most information was provided for the validity of the LSM questionnaires, more specifically for their construct validity showing predominantly hypothesis-confirming associations with physical, cognitive, psychosocial, financial, environmental, and/or sociodemographic variables. Given the overall complexity of real-life mobility [4], it was unexpected that only a few of the identified studies assessed the construct validity of the LSM questionnaires based on a conceptual framework of mobility including various potential determinants [21–23, 48]. Summarizing the results on construct validity of the same questionnaire across several studies, the UAB-LSA, the NHLSD, the LSA-CI and the interview/proxy-based LSA-IS were identified as the most valid questionnaires for the LSM construct. Test-retest reliability of the LSM questionnaires has also been evaluated quite frequently, with overall at least acceptable results. Thus, trained interviewers or proxies can achieve consistent results in stable persons, suggesting that the LSM questionnaires are easy to use and provide clear instructions for administration and scoring. Although it is essential that an assessment instrument can capture changes induced by intervention measures or long-term changes over time, sensitivity of change is clearly understudied, lacking for most LSM questionnaires. A possible reason for this might be the fact that the evaluation of this psychometric property requires a longitudinal study design with a repeated observation of LSM after a period of time in which a change has occurred (e.g., intervention-induced or time-related). The ability to detect such changes has only been demonstrated for the UAB-LSA, the LSA‑F, the LSA-CI, and the interview-based and proxy-based versions of the LSA-IS [15, 16, 21–23].

Among the different LSM questionnaires, the UAB-LSA stands out due to its widespread use and the availability of validated versions in various languages and populations. Potential limitations of the UAB-LSA are its relatively long, retrospective assessment period of 4 weeks and its focus on the community setting, which may complicate its use in studies focusing on more short-term LSM (changes), in populations with cognitive impairment due to possible recall bias or institutional settings. To promote recall of LSM, the recommended LSA-CI covers a shorter assessment period of 1 week and has shown to be feasible, valid, reliable and sensitive to intervention-induced changes in community-dwelling older persons with and without cognitive impairment [21, 48]. The two versions of the LSA-IS, which have been specifically developed for use in institutionalized older persons, even cover a shorter assessment period of 1 day, and have also demonstrated overall good psychometric properties in populations with and without cognitive impairment [22, 23]. A limitation of such short assessment period is that it does not cover day to day variability in LSM, although it can be assumed that variability in LSM tends to decrease in such settings due to institutional routines [51].

Overall, recommendations for use based on comprehensive results on adequate psychometric properties with high methodological quality could only be identified for the UAB-LSA [15] and the LSA-CI [32] for community-dwelling older persons (with cognitive impairment [21]), and the two versions (interview-based and proxy-based) of the LSA-IS for older persons staying in institutions [22, 23]. All four recommended LSM questionnaires are available in the German language. The German version of the UAB-LSA has been shown to be valid for measuring LSM but data on other psychometric properties in German populations are still lacking and should be investigated in the future. The LSA-CI and the interview-based and proxy-based LSA-IS versions have been comprehensively and successfully validated for use in German speaking older persons and the manuals and assessment forms are provided in the Supplemental Material to facilitate the application in clinical routine and research (Supplemental Documents 4–9).

Future studies on psychometric properties of LSM questionnaires should focus on feasibility aspects, select variables for construct validity analyses based on a conceptual LSM framework and evaluate the ability to detect changes in LSM. Future developments might combine the accuracy of sensor-based assessments with aspects evaluated by questionnaires concerning independence of mobility.

## Conclusion

Several life-space mobility (LSM) questionnaires are available for clinicians or researchers to assess spatial aspects of mobility in everyday life; however, only four questionnaires provided a comprehensive validation including good feasibility, validity, reliability and sensitivity to change. Given the comprehensive validation and overall good psychometric properties, the UAB-LSA and LSA-CI can be recommended for use in community-dwelling older persons and the two versions of the LSA-IS (interview-based and proxy-based) for use in institutionalized older persons. Each of these four LSM questionnaires is available in the German language.

## Practical conclusions

- Most identified LSM questionnaires showed acceptable to good psychometric properties, although validation strategies were partly limited to single psychometric properties in a number of studies.
- Based on the available evidence, we recommend the most comprehensively validated instruments with overall good psychometric properties: UAB-LSA and LSA-CI for community-dwelling older persons and the LSA-IS as self-based or proxy-based version for institutionalized older persons.
- All recommended questionnaires are validated for use in the German language.

## Supplementary Information

The supplemental documents include information on the search terms (Table S1), characteristics (Table S2) and psychometric properties (Table S3) of included assessment instruments, and the available assessment forms and manuals in German language (Supplemental documents S4–S9)

## Declarations

For this article no studies with human participants or animals were performed by any of the authors. The studies included in the review are subject to the ethical guidelines stated therein.
