# Supplementary material for: Assessing life-space mobility: A systematic review of questionnaires and their psychometric properties
Source: Z Gerontol Geriatr. 2022 Mar 4;55(8):660–6. doi: 10.1007/s00391-022-02035-5 (PMC9726808; doi:10.1007/s00391-022-02035-5)
Supplement: Supplementary file 1 — The supplemental documents include information on the search terms (Table S1), characteristics (Table S2) and psychometric properties (Table S3) of included assessment instruments, and the available assessment forms and manuals in German language (Supplemental documents S4–S9) [file 391_2022_2035_MOESM1_ESM.docx]

**Supplemental Documents**

**Table S1: Search Terms**

| *Title* | *Title/Abstract/Topic/All text (depending on database)* |
| --- | --- |
| *#1 Life-space* | *#5 psychometr** |
| *#2 Lifespace* | *#6 clinimetr** |
| *#3 “Life space”* | *#7 clinometr** |
| *#4 OR (#1-#3)* | *#8 measure** |
|  | *#9 valid** |
|  | *#10 assess** |
|  | *#11 instrument** |
|  | *#12 tool** |
|  | *#13 evaluat** |
|  | *#14 reproduc** |
|  | *#15 reliab** |
|  | *#16 unreliab** |
|  | *#17 coefficient* |
|  | *#18 homogen** |
|  | *#19 "internal consistency"* |
|  | *#20 correlation** |
|  | *#21 selection** |
|  | *#22 reduction** |
|  | *#23 test-retest* |
|  | *#24 inter** |
|  | *#25 kappa** |
|  | *#26 generali** |
|  | *#27 concordance* |
|  | *#28 discriminative* |
|  | *#29 factor** |
|  | *#30 subscale** |
|  | *#31 scaling* |
|  | *#32 error** |
|  | *#33 variability* |
|  | *#34 sensitiv** |
|  | *#35 responsive** |
|  | *#36 interpretab** |
|  | *#37 "meaningful change"* |
|  | *#38 ceiling* |
|  | *#39 floor* |
|  | *#40 Rasch* |
|  | *#41 cross-cultural* |
|  | *#42 Specific** |
|  | *#43 Feasib** |
|  | *#44 agreement* |
|  | *#45 precis** |
|  | *#46 imprecis** |
|  | *#47 repeatab** |
|  | *#48 replicab** |
|  | *#49 test AND retest* |
|  | *#50 cronbach* AND alpha** |
|  | *#51 limit AND detection* |
|  | *#52 (minimal* OR clinical* OR small* OR important OR significant OR detectable OR real) NEAR (change OR difference)* |
|  | *#53 OR (#5-#52)* |
|  | *#4 AND #53* |

**Table S2: Characteristics of the included assessment instruments**

| **Assessment instrument, author (reference), Country** | **Construct** | | **Persons in…** | |  | | **Administration** | | | | | **Aspects of assessment** | | | | | **Scoring** | |
| --- | --- | --- | --- | --- | --- | --- | --- | --- | --- | --- | --- | --- | --- | --- | --- | --- | --- | --- |
|  |  |  |  |  |  | | **Report** | | | **Equipment** | |  |  |  |  |  |  |  |
|  | **Life-Space Mobility** | **Life-Space** | **… the community** | **… in institutions** | **Self-report** | **Face-to-face** | | **Phone** | **Proxy-report** | **Specific requirements** | **Manual /tutorial** | **Recall period in days** | **Nr of Life-Space zones** | **Frequency (options)** | **Independence** | **Other aspects** | **Range of scores** | **Additional options** |
| Life-Space Diary (LSD) May et al. 1985 (May, Nayak, & Isaacs, 1985), Great Britain | 🗸 |  | 🗸 |  | 🗸^1^ |  | |  |  |  |  | 31 | 5 |  |  | weather + falls | 0-100 | 4 groups |
|  |  |  |  |  |  |  |  |  |  |  |  |  |  |  |  |  |  |  |
| Nursing Home Life-Space Diameter (NHLSD) Tinetti & Ginter 1990 (Tinetti & Ginter, 1990), USA | 🗸 |  |  | 🗸 |  |  | |  | 🗸 |  |  | 14 | 4 | 6 | 2 |  | 0-50 / 0-100 |  |
| Life-Space Questionnaire (LSQ) Stalvey et al. 1999 (Stalvey, Owsley, Sloane, & Ball, 1999), USA |  | 🗸 | 🗸 |  | 🗸 | 🗸 | |  |  |  | 🗸 | 3 | 10 |  |  |  | 0-9 |  |
| University of Alabama at Birmingham- Life-Space Assessment (UAB-LSA) Baker et al. 2003 (Baker, Bodner, & Allman, 2003), USA | 🗸 | 🗸 | 🗸 |  | 🗸 | 🗸 | | 🗸 |  |  | 🗸 | 28 | 6 | 4 | 3 |  | 0-120 | 4 sub-scores |
|  |  |  |  |  |  |  |  | ^2^ |  |  |  |  |  |  |  |  |  |  |
| (Phone-based) Life-Space Assessment (LSA-F) Auger et al. (Auger et al., 2009), Canada | 🗸 | 🗸 | 🗸 |  | 🗸 |  | |  |  |  |  | 28 | 6 | 4 | 3 |  | 0-120 | 4 sub-scores |
| Homebound Mobility assessment (HBMA) Allman et al. 2010 (Allman, Sawyer, Ritchie, Locher, & Brown, 2010), USA | 🗸 |  | 🗸 |  |  |  | | 🗸 | 🗸 |  |  | 1 | 5 |  | 3 |  | 0-8 | 3 groups |
| Indoor life-space mobility at home (LSH) Hashidate et al. 2013 (Hashidate et al., 2013), Japan | 🗸 |  | 🗸 |  | 🗸 | 🗸 | |  | 🗸 |  |  | 7 | 5 | 2 | 3 |  | 0-open end |  |
|  |  |  |  |  |  |  |  |  |  |  |  |  |  |  |  |  |  |  |
| Life-Space Assessment (LSA companion) Cavanaugh et al. 2014 (Cavanaugh & Crawford, 2014), USA | 🗸 |  | 🗸 |  |  | 🗸 | |  | 🗸 |  |  | 28 | 6 | 4 | 3 |  | 0-120 |  |
| Home-based life-space assessment (Hb-LSA) Ohnuma et al. 2014 (Ohnuma, Hashidate, Yoshimatsu, & Abe, 2014), Japan^3^ | 🗸 |  | 🗸 |  | 🗸 | 🗸 | |  |  |  |  | 31 | 5 | ? | ? |  | 0-120 |  |
| Life-Space Assessment for persons with cognitive impairment (LSA-CI) Ullrich et al. 2019 (Ullrich et al., 2019), Germany | 🗸 | 🗸 | 🗸 |  | 🗸 | 🗸 | |  |  |  | 🗸 | 7 | 6 | 3 | 3 |  | 0-90 | 3 sub-scores |
| Life-Space Assessment for Institutionalized Settings (LSA-IS) self-report (Hauer et al. 2020 (Hauer et al., 2020)), Germany | 🗸 | 🗸 |  | 🗸 | 🗸 | 🗸 | |  |  |  | 🗸 | 1 | 6 | 4 | 3 |  | 0-120 | 3 sub-scores |
| Map-based Life-space assessment Hinrichs et al. 2020 (Hinrichs et al., 2020), Switzerland |  | 🗸 | 🗸 |  | 🗸 | 🗸 | |  |  | 🗸 | 🗸 | 7 | 3 |  |  |  | 4 outcomes: 0-open |  |
| Life-Space Assessment for Institutionalized Settings (LSA-IS) proxy report Hauer et al. 2020 (Hauer et al., 2021)), Germany | 🗸 |  |  | 🗸 |  |  | |  | 🗸 |  | 🗸 | 1 | 6 | 4 | 3 |  | 0-120 | 3 sub-scores |

^1^via diary; ^2^Retest was conducted via phone; ^3^some information was extracted by Tanaka et al. (Tanaka & Yamagami, 2018); ^4^two for distance and two for area

**Table S3: Psychometric properties for the included assessment instruments**

| **Instrument** | **Language** | **Population** | **Psychometric properties** | | | | | | | | **Rating of methodology** | **Limitations with respect to results** |
| --- | --- | --- | --- | --- | --- | --- | --- | --- | --- | --- | --- | --- |
|  |  |  | **Feasibility** | **Validity** (V): Construct, Concurrent* or Content V, 🗸 if >=0.5 for similar, and >=0.3 for related constructs | Methodological limitations | **Reliability** (R): 🗸 if ICC/weighed kappa >0.70 | Methodological limitations | | **Sensitivity to change** | Methodological limitations | based on the COSMIN risk of bias checklist (Mokkink et al., 2018) |  |
| **LSD**  May et al. (1985), (May et al., 1985) | english | Community-dwelling older persons (mean age 77±7 years) | Completion rate 93%, no floor/ ceiling effects | n=30; Construct V: 2 significant out of 2 variables (for function), values🗸 | n of participants and construct variables |  |  | |  |  | V: moderate |  |
| **NHLSD**  Tinetti & Ginter (1990), (Tinetti & Ginter, 1990) | english | Nursing home residents (mean age 82 years) | No floor/ ceiling effects | n=398; Construct V: 5 significant out of 5 variables (for function + participation), values🗸 |  | n=25; Test-Retest + interrater R, results🗸 | Number of participants, statistical analysis (Pearson) | |  |  | V: high, R: moderate |  |
| (Tanaka & Yamagami, 2018) | japanese | Patients in geriatric health care facility (mena age 87±7 years) | No floor/ ceiling effects | n=32; Construct V: 8 significant out of 15 variables (for function, cognition, psychosocial status), values🗸; Concurrent V: with Hb-LSA value 🗸 | n of participants |  |  | |  |  | V: moderate | High n of non-significant correlations (Construct V) |
| **LSQ**  Stalvey et al. (1999), (Stalvey et al., 1999) |  | Community-dwelling older persons (age range 55-85 years) |  | n=242; Construct V: 8 significant out of 12 variables (for psychosocial status, function + environment), values low to 🗸 | 66% concordance with hypotheses | n=200; Test-Retest R: after 1 year, results 🗸 | Duration between test and retest | |  |  | V, R: moderate | partially low values (Construct V) |
| **UAB-LSA**  Baker et al. (2003) (Baker et al., 2003) | english | Community-dwelling older persons (mean age 75±7 years) | No floor/ ceiling effects | n=306; Construct V: 8 significant out of 8 variables (for function, psychosocial + demographic/ biographical status), values low to 🗸  Concurrent V in other studies |  | Test-Retest R: after 14 days, results🗸 | different test conditions | Changes over time: 10 pts. change | | No adequate method/ statistical analysis | V: high,  R: moderate,  S: low | partially low values (Construct V) |
| (Ji, Zhou, Liao, & Feng, 2015) | Chinese | Community-dwelling older persons (mean age 72±5 years) |  | n=100; Construct V: 6 significant out of 6 variables (for function, psychosocial status, cognition), values 🗸 |  | n=40, Test-Retest R: after 14 days, results🗸 | n of participnats, different test conditions |  | |  | V: high, R: moderate |  |
| (Tseng, Gau, & Lou, 2020) | Chinese | Community-dwelling older persons (mean age 73±7 years) | Assessment duration 3-5 minutes | n=225; Construct V: 3 significant out of 3 variables (for function, psychosocial, status), values 🗸; Content V: index (I-CVI) and a scale-level content validity index 🗸 | n of construct variables; no patients included for Content V | Test-Retest R: after 14 days, results🗸; Interrater R: results 🗸 |  |  | |  | V: moderate, R: high |  |
| (Pedersen, Kjaer-Sorensen, Midtgaard, Brown, & Bodilsen, 2019) | Danish | Community-dwelling older persons (mean age 79 years) |  | n=30; Content V: 🗸 (focus translation) |  |  |  |  | |  | V: high |  |
| (Portegijs, Iwarsson, Rantakokko, Viljanen, & Rantanen, 2014) | Finnish | Community-dwelling older persons (mean age 81±4) | No floor/ ceiling effects |  |  | n=41; Test-Retest R: after 14 days (winter-spring) 🗸 | n of participnats | n=808, anchor-based method: results🗸 | |  | R: moderate, S: high |  |
| (Mümken, Gellert, Stollwerck, O'Sullivan, & Kiselev, 2021) | German | Community-dwelling older persons (mea n age 79±5 years) |  | n=83; Construct V: 6 significant out of 8 variables (for function, psychosocial, status, demographic data/biographical status) values 🗸 |  |  |  |  | |  | V: high |  |
| (Harada et al., 2010) | Japanese | Community-dwelling older persons (mean age 79±7 years) |  | n=2147; Construct V: 4 significant out of 4 variables (for function, demographic data/biographical status) values low to 🗸 |  |  |  |  | |  | No rating (only abstract in english language) | partially low values (Construct V) |
| (Ferreira, Cavalheiro, Fernandes, Goncalves, & Ferreira, 2018) | Portuguese | Community-dwelling older persons (mean age 74±7 years) |  | n=520; Construct V: 15 significant out of 15(?) variables (for function, psychosocial, status, demographic data/biographical status) values low to 🗸 |  | n=103; Test-Retest R: after 7 days, results 🗸 |  | n=154; with intervention: SRM 0.40 | |  | No rating (only conference abstract) | Low value (S) |
| (Simões, Garcia, Costa, & Lunardi, 2018) | Portuguese | Community-dwelling older persons (mean age 70±9 years) | No floor/ ceiling effects | n=80; Construct V: 2 significant out of 2 variables (for function) values 🗸; Content V: 🗸 | n of construct variables | Test-Retest R: after 7 days, results 🗸 |  |  | |  | V: moderate, R: high |  |
| (Curcio et al., 2013) | Portuguese and Spanish | Community-dwelling older persons (age range 65-74) |  | n=300; Construct V: 10 significant out of 11 variables (for function, cognition, psychosocial + demographic / biographical, financial status) values 🗸 |  | n=39; Test-Retest R: after 7-10 days, results 🗸 | n=39, only spanish version |  | |  | V: high, R: moderate |  |
| (Fristedt, Kammerlind, Bravell, & Fransson, 2016) | Swedish | Community-dwelling older persons (mean age 80±5 years) |  | n=327; Construct V: 7 significant out of 7 variables (function) values 🗸 (reported as concurrent validity in the article) |  |  |  |  | |  | V: high |  |
| (Kammerlind, Fristedt, Ernsth Bravell, & Fransson, 2014) | Swedish | Community-dwelling older persons (mean age 80±5 years) |  |  |  | n=298; Test-Retest R: after 14 days, results 🗸 | Different test conditions |  | |  | R: moderate |  |
| (Alshebber, Dunlap, & Whitney, 2020) | english | Persons with Vestibular disorders (mea nage 55±17) |  | n=128; Construct V: 6 significant out of 6 variables (function, psychosocial status) values 🗸 |  | n=37; Test-Retest R: after several hours, results 🗸 | n of participants |  | |  | V: high, R: moderate |  |
| (Zhu et al., 2020) | english | Persons with Parkinson disease (mean age 68±6 years) |  | n=54; Concurrent V: GPS-data, values low-moderate | Focus on validation of GPS assessment |  |  |  | |  | V: moderate | low value (Concurrent V) |
| (Iyer et al., 2017) | english | Persons with Chronic obstructive pulmonary disease (mean age 66±10 years) |  | n=47: Construct V: 4 significant out of 4 variables (for function, psychosocial status, health) values 🗸 |  |  |  |  | |  | No rating (only conferene abstract) |  |
| (Petti, Hope, Hsieh, Hurtado-Sbordoni, & Gong, 2015) | english | Persons with Critical Illness | Reported without values | n=35 (patients) / 49 (proxys): Construct V: no detailed results reported | No values given |  |  |  | |  | No Rating (only conference abstract) |  |
| (Phillips, Lam, Luckett, Agar, & Currow, 2014) | english | Persons in palliative care (mean age 75±10 years) |  | n=98; Construct V: 9 significant out of 17 variables (for function, psychosocial status) values low to 🗸 | Part of non-significant correlations high due to consideration of all subscores |  |  |  | |  | V: high | Partially low values for less associated variables |
| (Yang et al., 2017) | Korean | Persons with stroke (mean age 65±2 years) |  | n=34; Construct V: 5 significant out of 5 variables (for function, psychosocial status) 🗸 | n of participants | Test-Retest R: after 14 days, results 🗸 | n of participnats |  | |  | V, R: moderate |  |
| (Estima, Dutra, Martins, & Franzoi, 2015) | Portuguese | patients with stroke (mean age 59 years) | No floor/ ceiling effects | n=30; Construct V: 5 significant out of 7 variables (for function, cognition,demographic status) values 🗸 | n of participants | Test-Retest R: after ? days; Interrater R, results 🗸 | n of participnats |  | |  | V, R: moderate |  |
| (I. Garcia, Tiuganji, Simoes, Santoro, & Lunardi, 2016; I. F. F. Garcia, Tiuganji, Simoes, & Lunardi, 2018) | Portuguese | Persons with Chronic obstructive pulmonary disease (mean age 65/77±4 years) | No floor/ ceiling effects | n=62; Construct V: 1 significant out of 1 variable (for function) values🗸 | n of construct variables | Test-Retest R: after 7 days, results 🗸 |  |  | |  | V: moderate, R: high |  |
| **LSA – via phone**  Auger et al. (Auger et al., 2009) | French (-canadian) | Persons with power mobility devices (age range 45-82) | Completion rate 92%; no floor/ ceiling effects; assessment duration 9 minutes | n=5; Content validity:  86% agreement (not rated), focus translation🗸 | Only one patient included | n=40, Test-Retest R: after 14 days, results 🗸 | n of participnats |  | |  | V, R: moderate |  |
| via phone (Lanzino et al., 2016) | english | Persons with spinal cord injury (mean age 46 ±13 / 43±14 years) | No floor/ ceiling effects | n=50; Construct V: 3 significant out of 3 variables (for function, psychosocial status, participation) values 🗸 | n of construct variables | Test-Retest R: after 9 days, results 🗸 |  |  | |  | V: moderate, R: high |  |
| via phone (McCrone, Smith, Hooper, Parker, & Peters, 2019) | english | Persons undergoing community-based physical therapy intervention (mean age 81 years; age range 25-99 years) | Completion rate 82.6%; no floor/ ceiling effects | n=276; Construct V: 1 significant out of 1 variable (function) values low | n of construct variables |  |  | n=228; with intervention (after 53 days): significant change of 10.5 points; subgroup comparison | | No adequate statistical aproach | V: low, S: moderate | Low values |
| **HBMA**, Allman et al. (Allman et al., 2010) | english | Community-dwelling older persons (mean age 85±7 years) |  | n=53; Construct V: 3 significant out of 3 variables (for function) values 🗸 | n of construct variables |  |  |  | |  | V: moderate |  |
| **LSH**, Hashidate et al. (Hashidate et al., 2013) | Japanese | Community-dwelling older persons (mean age 77±5 years) |  | n=20; Construct V: 6 significant out of 7 variables (for function, cognition) values 🗸;  Concurrent V: with UAB-LSA; ICC=0.33 | n of participants | Test-Retest R: after up to 7 days, results 🗸 | n of participants |  | |  | V, R: moderate | low value (concurrent V) |
| **LSA companion**,  Cavanaugh et al. (Cavanaugh & Crawford, 2014) | english | Community-dwelling older persons (mean age 81±8 years) |  | n=40; Concurrent V: with UAB-LSA value 🗸 | n of participants |  |  |  | |  | V: moderate |  |
| **Hb-LSA**,  Ohnuma et al. (Ohnuma et al., 2014) | Japanese | Community-dwelling older persons (mean age 79±7 years) | No ceiling/ floor effects | n=37; Construct V: 5 significant variables out of 7 variables (for function) values 🗸 (Concurrent V in (Tanaka & Yamagami, 2018)) | n of participants | Test-Retest R: after ? days, results 🗸 | n of participants |  | |  | No rating (only abstract in english language) |  |
| **LSA-CI**,  Ullrich et al. (Ullrich et al., 2019) | German | Community-dwelling older adults with CI (mean age 82±6 years) | Completion rate 100%; no floor/ ceiling effects; assessment duration 4 minutes | n=117; Construct V: 16 significant out of 20 variables (based on mobility model**) values 🗸 |  | n=102; Test-Retest R: after 1 day, results 🗸 |  | n=52; with intervention, SRM 0.80 | |  | V, R,  S: high*** |  |
| (Ullrich, Abel, Bauer, & Hauer, 2021) | German | Community-dwelling older adults without CI (mean age 81±6 years) | Completion rate 100%; no floor/ ceiling effects; assessment duration 4 minutes | n=65; Construct V: 8 significant out of 9 variables (for function, physical activity, psychosocial and demographic status) values 🗸 |  | n=55; Test-Retest R: after 1 day, results 🗸 |  | n=32; with intervention, SRM 0.70 | | n of participants | V, R: high,  S: moderate*** |  |
| **LSA-IS self-report**,  Hauer et al. (Hauer et al., 2020) | German | Institutionalized older persons (mean age 83±6 years) | Completion rate 100%; no floor/ ceiling effects; assessment duration 3 minutes | n=119; Construct V: 15 significant out of 18 variables (based on mobility model**) values 🗸 |  | n=76; Test-Retest R: after 1 day, results 🗸 |  | n=69; with intervention, SRM 0.81 | |  | V, R,  S: high^***^ |  |
| **Map-based Life-space assessment**,  Hinrichs et al. (Hinrichs et al., 2020) | German (Suisse) | Community-dwelling older adults (mean age 74±6 years) |  | n=58 (valid n=37); Concurrent V: with GPS-data, values low to 🗸 | n of participants | N=56/57; Test-Retest R: after 22.3 days, results - |  |  | |  | V: moderate, R: high | Overall low values (V,R) |
| **LSA-IS proxy report**,  Hauer et al. (Hauer et al., 2021) | German | Institutionalized older persons (mean age 83±6 years) | Completion rate 100%; no floor/ ceiling effects | n=94, Construct V: 10 significant out of 14 variables (based on mobility model**) values 🗸; Concurrent V: with proxy report values 🗸 |  | n= 85; Test-Retest R: after 1 day, results 🗸 |  | n=69; with intervention, SRM 0.44 | | Intervetion not adequately described | V, R: high, S: moderate******* | Low value (S) |

* Validity studies were only classified as “Concurrent validity” if other assessment instruments including spatial aspects of mobility (LSM or GPS-based assessment) were used for comparison

**Variable selection based on a theory driven mobility model [4], including the domains cognitive, psychosocial, physical, environmental, and financial status and gender/cultural/biographical influences

*** Quality rating by BA and MH

Abbreviations: r=correlation coefficient (Spearman, Pearson or bivariate); ICC= Intraclass Correlation Coefficient; n=number; SRM= standardized response means

References

Allman, R. M., Sawyer, P., Ritchie, C. S., Locher, J. L., & Brown, C. J. (2010). *Preliminary Validation of a Telephone Assessment of Mobility for Homebound Older Adults (B79)*. Paper presented at the J Am Geriatr Soc. <https://agsjournals.onlinelibrary.wiley.com/doi/abs/10.1111/j.1532-5415.2010.02850.x>

Alshebber, K. M., Dunlap, P. M., & Whitney, S. L. (2020). Reliability and Concurrent Validity of Life Space Assessment in Individuals With Vestibular Disorders. *J Neurol Phys Ther, 44*(3), 214-219. doi:10.1097/npt.0000000000000320

Auger, C., Demers, L., Gélinas, I., Routhier, F., Jutai, J., Guérette, C., & Deruyter, F. (2009). Development of a French-Canadian version of the Life-Space Assessment (LSA-F): content validity, reliability and applicability for power mobility device users. *Disabil Rehabil Assist Technol, 4*(1), 31-41. doi:10.1080/17483100802543064

Baker, P. S., Bodner, E. V., & Allman, R. M. (2003). Measuring life-space mobility in community-dwelling older adults. *J Am Geriatr Soc, 51*(11), 1610-1614. doi:10.1046/j.1532-5415.2003.51512.x

Cavanaugh, J. T., & Crawford, K. (2014). Life-Space Assessment and Physical Activity Scale for the Elderly: validity of proxy informant responses. *Arch Phys Med Rehabil, 95*(8), 1527-1532. doi:10.1016/j.apmr.2014.03.027

Curcio, C. L., Alvarado, B. E., Gomez, F., Guerra, R., Guralnik, J., & Zunzunegui, M. V. (2013). Life-Space Assessment scale to assess mobility: validation in Latin American older women and men. *Aging Clin Exp Res, 25*(5), 553-560. doi:10.1007/s40520-013-0121-y

Estima, A., Dutra, B. M. T., Martins, J. V., & Franzoi, A. (2015). *Validation of the@ Life Space Assessment - LSA Questionnaire in a group of hemiplegic patients*.

Ferreira, L. S., Cavalheiro, L., Fernandes, F., Goncalves, R. S., & Ferreira, P. L. (2018). Life-space assessment scale to assess Portuguese older adults mobility: cross-cultural adaptation and validation. *Quality of Life Research, 27*, S67-S67.

Fristedt, S., Kammerlind, A. S., Bravell, M. E., & Fransson, E. I. (2016). Concurrent validity of the Swedish version of the life-space assessment questionnaire. *BMC Geriatr, 16*(1), 181. doi:10.1186/s12877-016-0357-4

Garcia, I., Tiuganji, C., Simoes, M. D., Santoro, I., & Lunardi, A. C. (2016). Measurement proprieties of University of Alabama at Birmingham study of aging life-space assessment questionnaire in elderly with COPD. *European Respiratory Journal, 48*. doi:10.1183/13993003.congress-2016.PA4444

Garcia, I. F. F., Tiuganji, C. T., Simoes, M., & Lunardi, A. C. (2018). A study of measurement properties of the Life-Space Assessment questionnaire in older adults with chronic obstructive pulmonary disease. *Clinical Rehabilitation, 32*(10), 1374-1382. doi:10.1177/0269215518780488

Harada, K., Shimada, H., Sawyer, P., Asakawa, Y., Nihei, K., Kaneya, S., . . . Yasumura, S. (2010). [Life-space of community-dwelling older adults using preventive health care services in Japan and the validity of composite scoring methods for assessment]. *Nihon Koshu Eisei Zasshi, 57*(7), 526-537.

Hashidate, H., Shimada, H., Shiomi, T., Shibata, M., Sawada, K., & Sasamoto, N. (2013). Measuring indoor life-space mobility at home in older adults with difficulty to perform outdoor activities. *J Geriatr Phys Ther, 36*(3), 109-114. doi:10.1519/JPT.0b013e31826e7d33

Hauer, K., Ullrich, P., Heldmann, P., Bauknecht, L., Hummel, S., Abel, B., . . . Werner, C. (2021). Psychometric Properties of the Proxy-Reported Life-Space Assessment in Institutionalized Settings (LSA-IS-Proxy) for Older Persons with and without Cognitive Impairment. *Int J Environ Res Public Health, 18*(8). doi:10.3390/ijerph18083872

Hauer, K., Ullrich, P., Heldmann, P., Hummel, S., Bauer, J. M., & Werner, C. (2020). Validation of the interview-based life-space assessment in institutionalized settings (LSA-IS) for older persons with and without cognitive impairment. *BMC Geriatr, 20*(1), 534. doi:10.1186/s12877-020-01927-8

Hinrichs, T., Zanda, A., Fillekes, M. P., Bereuter, P., Portegijs, E., Rantanen, T., . . . Weibel, R. (2020). Map-based assessment of older adults' life space: validity and reliability. *Eur Rev Aging Phys Act, 17*(1), 21. doi:10.1186/s11556-020-00253-7

Iyer, A. S., Jones, A., Kirkpatrick, D. P., Nichols, J., Wells, J. M., Bhatt, S. P., . . . Dransfield, M. (2017). Life Space Assessment As A Measure Of Community Mobility In Chronic Obstructive Pulmonary Disease. *American Journal of Respiratory and Critical Care Medicine, 195*.

Ji, M., Zhou, Y., Liao, J., & Feng, F. (2015). Pilot study on the Chinese version of the Life Space Assessment among community-dwelling elderly. *Arch Gerontol Geriatr, 61*(2), 301-306. doi:10.1016/j.archger.2015.06.012

Lanzino, D., Sander, E., Mansch, B., Jones, A., Gill, M., & Hollman, J. (2016). Life Space Assessment in Spinal Cord Injury. *Top Spinal Cord Inj Rehabil, 22*(3), 173-182. doi:10.1310/sci2203-173

May, D., Nayak, U. S., & Isaacs, B. (1985). The life-space diary: a measure of mobility in old people at home. *Int Rehabil Med, 7*(4), 182-186. doi:10.3109/03790798509165993

McCrone, A., Smith, A., Hooper, J., Parker, R. A., & Peters, A. (2019). The Life-Space Assessment Measure of Functional Mobility Has Utility in Community-Based Physical Therapist Practice in the United Kingdom. *Phys Ther, 99*(12), 1719-1731. doi:10.1093/ptj/pzz131

Mokkink, L. B., de Vet, H. C. W., Prinsen, C. A. C., Patrick, D. L., Alonso, J., Bouter, L. M., & Terwee, C. B. (2018). COSMIN Risk of Bias checklist for systematic reviews of Patient-Reported Outcome Measures. *Quality of Life Research, 27*(5), 1171-1179. doi:10.1007/s11136-017-1765-4

Mümken, S. A., Gellert, P., Stollwerck, M., O'Sullivan, J. L., & Kiselev, J. (2021). Validation of the German Life-Space Assessment (LSA-D): cross-sectional validation study in urban and rural community-dwelling older adults. *BMJ Open, 11*(7), e049926. doi:10.1136/bmjopen-2021-049926

Ohnuma, T., Hashidate, H., Yoshimatsu, T., & Abe, T. (2014). [Clinical usefulness of indoor life-space assessment in community-dwelling older adults certified as needing support or care]. *Nihon Ronen Igakkai Zasshi, 51*(2), 151-160. doi:10.3143/geriatrics.51.151

Pedersen, M. M., Kjaer-Sorensen, P., Midtgaard, J., Brown, C. J., & Bodilsen, A. C. (2019). A Danish version of the life-space assessment (LSA-DK) - translation, content validity and cultural adaptation using cognitive interviewing in older mobility limited adults. *BMC Geriatr, 19*(1). doi:10.1186/s12877-019-1347-0

Petti, A., Hope, A. A., Hsieh, S. J., Hurtado-Sbordoni, M., & Gong, M. N. (2015). Feasibility and Validity of Life Space Mobility Assessment in Critically Ill Adults. *J Am Geriatr Soc, 63*, S250-S251.

Phillips, J. L., Lam, L., Luckett, T., Agar, M., & Currow, D. (2014). Is the Life Space Assessment applicable to a palliative care population? Its relationship to measures of performance and quality of life. *J Pain Symptom Manage, 47*(6), 1121-1127. doi:10.1016/j.jpainsymman.2013.06.017

Portegijs, E., Iwarsson, S., Rantakokko, M., Viljanen, A., & Rantanen, T. (2014). Life-space mobility assessment in older people in Finland; measurement properties in winter and spring. *BMC Res Notes, 7*, 323. doi:10.1186/1756-0500-7-323

Simões, M., Garcia, I. F., Costa, L. D. C., & Lunardi, A. C. (2018). Life-Space Assessment questionnaire: Novel measurement properties for Brazilian community-dwelling older adults. *Geriatr Gerontol Int, 18*(5), 783-789. doi:10.1111/ggi.13263

Stalvey, B. T., Owsley, C., Sloane, M. E., & Ball, K. (1999). The Life Space Questionnaire: A measure of the extent of mobility of older adults. *Journal of Applied Gerontology, 18*(4), 460-478. doi:10.1177/073346489901800404

Tanaka, S., & Yamagami, T. (2018). Life-space and Related Factors for the Elderly in a Geriatric Health Service Facility. *Prog Rehabil Med, 3*, 20180001. doi:10.2490/prm.20180001

Tinetti, M. E., & Ginter, S. F. (1990). The nursing home life-space diameter. A measure of extent and frequency of mobility among nursing home residents. *J Am Geriatr Soc, 38*(12), 1311-1315. doi:10.1111/j.1532-5415.1990.tb03453.x

Tseng, Y. C., Gau, B. S., & Lou, M. F. (2020). Validation of the Chinese version of the Life-Space Assessment in community-dwelling older adults. *Geriatr Nurs, 41*(4), 381-386. doi:10.1016/j.gerinurse.2019.11.014

Ullrich, P., Abel, B., Bauer, J. M., & Hauer, K. (2021). Validation of the Life-Space Assessment (LSA-CI) in multi-morbid, older persons without cognitive impairment. *Eur Geriatr Med, 12*(3), 657-662. doi:10.1007/s41999-020-00441-9

Ullrich, P., Werner, C., Bongartz, M., Kiss, R., Bauer, J., & Hauer, K. (2019). Validation of a Modified Life-Space Assessment in Multimorbid Older Persons With Cognitive Impairment. *Gerontologist, 59*(2), e66-e75. doi:10.1093/geront/gnx214

Yang, Y. N., Kim, B. R., Uhm, K. E., Kim, S. J., Lee, S., Oh-Park, M., & Lee, J. (2017). Life Space Assessment in Stroke Patients. *Ann Rehabil Med, 41*(5), 761-768. doi:10.5535/arm.2017.41.5.761

Zhu, L., Duval, C., Boissy, P., Montero-Odasso, M., Zou, G., Jog, M., & Speechley, M. (2020). Comparing GPS-Based Community Mobility Measures with Self-report Assessments in Older Adults with Parkinson's Disease. *J Gerontol A Biol Sci Med Sci, 75*(12), 2361-2370. doi:10.1093/gerona/glaa012

**Supplemental Document 4: LSA-CI Assessment Form (in German language)**

**LSA-CI**

| Name: | | | Datum: | | |
| --- | --- | --- | --- | --- | --- |
| Diese Fragen beziehen sich auf Ihre Aktivitäten innerhalb der **letzten Woche**: | | | | | |
| **Lebensraum** | | **Häufigkeit** | | **Selbstständigkeit** | **Punktzahl** |
| Waren Sie während der letzten Woche… | | Wie oft waren Sie dort? | | Haben Sie Hilfsmittel benötigt? Haben Sie die Hilfe einer anderen Person benötigt? | Stufe x  Häufigkeit x Selbstständigkeit |
| Stufe 1:…in anderen Räumen bei Ihnen zu Hause außer Ihrem Schlafzimmer? | 1 = Ja  0 = Nein | 1 = 1-3 Mal/ Woche  2 = 4-6 Mal/ Woche  3 = Täglich | | 1 = persönliche Hilfe  1,5 = Nur Hilfsmittel  2 = Ohne Hilfe | _________  Stufe 1 Punkte |
| Punktzahl (multiplizieren) | ____ x | _______x | | _______ = |  |
| Stufe 2:…außerhalb Ihrer Wohnung, wie z.B. ihrer/m Veranda, Terrasse oder Hof, Hausflur oder Garage, Garten oder Auffahrt? | 2 = Ja  0 = Nein | 1 = 1-3 Mal/ Woche  2 = 4-6 Mal/ Woche  3 = Täglich | | 1 = Hilfsperson  1,5 = Nur Hilfsmittel  2 = Ohne Hilfe | _________  Stufe 2 Punkte |
| Punktzahl (multiplizieren) | ____ x | _______ x | | _______ = |  |
| Stufe 3:…in Ihrer Nachbarschaft außerhalb Ihres Wohnhauses oder Gartens? | 3 = Ja  0 = Nein | 1 = 1-3 Mal/ Woche  2 = 4-6 Mal/ Woche  3 = Täglich | | 1 = Hilfsperson  1,5 = Nur Hilfsmittel  2 = Ohne Hilfe | _________  Stufe 3 Punkte |
| Punktzahl (multiplizieren) | ____ x | _______ x | | _______ = |  |
| Stufe 4:…außerhalb Ihrer Nachbarschaft, aber innerhalb Ihres Wohnortes? | 4 = Ja  0 = Nein | 1 = 1-3 Mal/ Woche  2 = 4-6 Mal/ Woche  3 = Täglich | | 1 = Hilfsperson  1,5 = Nur Hilfsmittel  2 = Ohne Hilfe | _________  Stufe 4 Punkte |
| Punktzahl (multiplizieren) | ____ x | _______ x | | _______ = |  |
| Stufe 5:…außerhalb Ihres Wohnortes? | 5 = Ja  0 = Nein | 1 = 1-3 Mal/ Woche  2 = 4-6 Mal/ Woche  3 = Täglich | | 1 = Hilfsperson  1,5 = Nur Hilfsmittel  2 = Ohne Hilfe | _________  Stufe 5 Punkte |
| Punktzahl (multiplizieren) | ____ x | _______x | | _______ = |  |
| **Gesamtpunktzahl (Summe Level 1 – Level 5)** | | | | | _________ |

**Supplemental Document 5: LSA-CI Assessment Manual (in German language)**

**Life-Space Assessment for Persons with Cognitive Impairment (LSA-CI)**

**–**

**Manual für Nutzer**

**Einführung**

Das “Life-Space Assessment for Persons with Cognitive Impairment” (LSA-CI) wurde auf Basis des “University of Alabama at Birmingham – Life Space Assessment” (UAB-LSA; Baker et al. 2003) entwickelt, um die “Life-Space Mobilität” auch von älteren Menschen mit kognitiver Einschränkung erfassen zu können. Es wurden Anpassungen und Veränderungen im Hinblick auf das eventuell eingeschränkte Erinnerungsvermögen bei dieser besonders vulnerablen Personengruppe vorgenommen. Aus diesem Grund wurde die Observationsdauer von vier Wochen auf eine Woche reduziert und eine spezielle Interviewtechnik eingeführt, die eine verzerrte Erfassung der Mobilität durch fehlendes Erinnerungsvermögen verhindern soll. Diese wurde auch schon erfolgreich in früheren Projekten (einem Fragebogen zur Erfassung der körperlichen Aktivität bei Personen mit leichter bis moderater kognitiver Einschränkung) eingesetzt (Hauer et al. 2011).

**Zielpopulation**

Die Zielpopulation umfasst ältere Menschen mit beginnender bis moderater kognitiver Einschränkung (basierend auf Mini-Mental Status Examination (MMSE), bei Ergebnissen zwischen 17-26 Punkten). Diese Personen zeigen häufig krankheitsbedingte Symptome wie Verlust des Erinnerungsvermögens, Wortfindungsstörungen, Sprechstörungen, eingeschränkte räumlich-zeitliche Orientierung, Einschränkungen der visuellen Wahrnehmung oder der Aufmerksamkeit (Knopman & Petersen 2014). Dies kann zu Verzerrungen der Erinnerung und Ungenauigkeiten bei dem Berichten zurückliegender Ereignisse (Shephard, 2003) und Schwierigkeiten beim Berichten der eigenen körperlichen Aktivität (Bhandari & Wagner, 2006; Sallis & Saelens, 2000) führen.

**Bestandteile des LSA-CI**

Der LSA-CI beinhaltet Fragen zum Betreten bzw. Aufenthalt in fünf verschiedenen “Life-Space“ Zonen (0 = Schlafzimmer [welches nur indirekt erfasst wird]; 1 = eigene Wohnung oder Haus; 2 = direkte Umgebung des Wohnhauses (Terrasse, Hof, Garten); 3 = Nachbarschaft; 4 = Stadt, 5= außerhalb der Stadt), zur Häufigkeit des Betretens bzw. Aufenthalts in der jeweiligen Zone (täglich, 4-6 x/Woche, 1-3x/Woche), und zum Bedarf an Hilfe oder Hilfsmitteln (ohne Hilfsmittel/-person, mit Hilfsmittel, mit Hilfsperson). Die Konzeption des Fragebogens stimmt mit dem Original UAB-LSA überein, abgesehen von der Einteilung und Bewertung der Häufigkeit, was an der reduzierten Observationsdauer (eine statt vier Wochen) liegt.

**Vorbereitungen für die Durchführung des LSA-CI**

Für Personen in Gesundheitsberufen ist kein spezielles Training erforderlich um den LSA-CI durchzuführen. Benötigt werden lediglich der Fragebogen und ein Stift.

**Vorgehen bei der Durchführung**

Der LSA-CI sollte interview-basiert durchgeführt wurden und die speziellen Bedürfnisse von Personen mit kognitiver Einschränkung berücksichtigen. Um die Aufmerksamkeit der zu befragenden Person nicht zu beeinträchtigen, sollte die Erhebung alleine stattfinden. Sollten Angehörige/Pflegekräfte anwesend sein, dürfen diese nicht das Antworten übernehmen oder sich einmischen. Im Anschluss an die Durchführung können die Angehörigen/Pflegekräfte jedoch die Aussagen bestätigen oder falsifizieren.

Das Befragung sollte in einem ruhigen Umfeld durchgeführt werden, am besten in der Wohnung des Befragten, so kann sich der Interviewer sich auf die Wohnsituation beziehen und die Fragen konkret stellen (zum Beispiel bei Life-Space Zone 1 und 2 gezielt Haus / Garten / Terrasse / Garten / Hof / Garage etc. erwähnen, bei Life-Space Zone 3 und 4 gezielt die Örtlichkeiten in der Nachbarschaft oder Stadt ansprechen, wie Supermärkte, Parks, Plätze, Briefkasten, Stationen des öffentlichen Nahverkehrs, Arztbesuche, etc.), oder bei Life-Space Zone 5 berücksichtigen, ob es sich um eine Stadt oder ländliche Region (relevant für Einkaufsmöglichkeiten, Arztbesuche etc.). Das Wissen um die aktuelle Wohnsituation ist auch hilfreich, um die Genauigkeit der Antworten direkt erfassen zu können und gegebenenfalls Rückfragen zu stellen (z.B. steile Treppe am Eingangsbereich, Stellplatz für Hilfsmittel etc.). Es sollte gezielt nach offensichtlichen Einschränkungen in der Beweglichkeit (z.B. Schwierigkeiten beim Aufstehen, Treppengehen etc.) und nach Hilfsmittel (z.B. sichtbarer Gehstock, Rollator etc.) gefragt werden, um einen realistischen Eindruck der aktuellen Fähigkeiten und Tätigkeiten zu erlangen.

***Demenzspezifische Interview-Technik:***

Die Befragung sollte im persönlichen Gespräch und interviewbasiert erfolgen, um Ungenauigkeiten auszuschließen bzw. zu reduzieren, die Vollständigkeit der Antworten abzusichern und gleichzeitig auf eine mögliche Überförderung des Befragten eingehen zu können (Hauer et al. 2009). Diese Form der Erfassung erlaubt umfassende mündliche Erläuterungen und lässt Raum für Rückfragen, was Ungenauigkeiten oder Mehrdeutigkeit verhindert (Durante & Ainsworth 1996) und Versagensängste in Bezug auf Verständnis und Erinnerungsfähigkeit bei den Befragten verhindern (Hauer et al 2011).

**Schritt 1:** Die Befragung sollte mit einer kurzen Erläuterung der Ziele und der Dauer der Befragung (insgesamt etwa 3 bis 5 Minuten) starten.

**Schritt 2:** Wichtig ist, den Beginn und das Ende des Beobachtungszeitraums klar zu benennen (z.B. Dienstag vor einer Woche bis jetzt). Falls während der Befragungen Unklarheiten auftreten, kann es angemessen sein, die Aktivitäten in jeder Zone für jeden Tag einzeln abzufragen.

**Schritt 3:** Fragen Sie einzeln nach jeder Life-Space Zone, beginnend mit Zone 1 und dann kontinuierlich weitergehend ohne eine Zone auszulassen.

Spezielle Zeitfenster sowie tägliche oder wöchentliche Rituale und Gewohnheiten können dabei als “Anker” genutzt werden, um den Beobachtungszeitraum zu segmentieren oder einzuteilen ( z.B. Wochenende und Wochentage, Aufstehen, Mahlzeiten, Körperpflege, kurze Einkäufe, Gang zum Briefkasten/Bäcker, Kiosk, Gartenarbeiten, Fernsehen, Putzen, Wäsche machen, Sportgruppen, Tagespflege, kirchliche Aktivitäten etc.) (Baranowski 1988).

Zusätzlich sollte gezielt nach typischen Aktivitäten gefragt werden, sofern diese nicht selbst erwähnt werden (Einkaufen, Arztbesuche, Therapien), oder besondere Aktivitäten wie Besuche von Freunden /Familie, Feste, Ausflüge etc. um die Vollständigkeit der Erfassung abzusichern. Geschlossene Fragen können dabei besser geeignet sein um eine kognitive Überforderung zu vermeiden (“Waren sie einkaufen” statt “Was haben Sie sonst gemacht?”).

Im Fokus der Erfassung steht die aktuelle Mobilität, also das, was die Befragten tatsächlich in der letzten Woche gemacht haben, und nicht das, wozu sie in der Lage gewesen wären.

Ein Anhaltspunkt für die Genauigkeit der Erfassung der einzelnen Zonen ist, dass es nicht möglich ist, eine Zone zu erreichen, ohne sich in den niedrigeren Zonen bewegt zu haben (z.B. ist es nicht möglich, in der Stadt (Zone 4) unterwegs gewesen zu sein, ohne die Nachbarschaft (Zone 3) durchquert zu haben.

Es wird keine exakte Einteilung bzw. Grenze für die Nachbarschaft oder Stadt vorgegeben, die Antworten, die die Befragten geben, werden so akzeptiert.

**Schritt 4:** Die Befragung schließt mit einer Zusammenfassung des Gesagten für jede Zone einschließlich der Häufigkeit und des Bedarfs an Hilfe, so haben die Befragten Zeit, ihre eigenen Aussagen zu prüfen und zu bestätigen oder zu korrigieren.

**Beispiele für die Befragung und die Zusammenfassung:**

Zone 1: *Haben Sie sich in der vergangenen Woche in ihrer Wohnung / Ihrem Haus außerhalb Ihres Schlafzimmers aufgehalten? Verlassen Sie nach dem Aufstehen jeden Morgen ihr Bett? Waren Sie in der vergangenen Woche krank und haben einen oder mehrere Tage im Bett verbracht? Benötigen Sie Hilfe, wenn Sie Ihr Bett verlassen, halten Sie sich an Ihrem Rollator/ der Kommode etc. fest? Wenn Sie in das Bett oder die Küche gehen, nutzen Sie dann den Rollator/Gehstock?*

Zusammenfassung Zone 1: *Also, Sie haben in der vergangenen Woche jeden Tag ihr Bett verlassen und dabei keine Hilfsmittel benötigt? Ist das so korrekt?*

Zone 2: *Waren Sie in der vergangenen Woche außerhalb Ihrer Wohnung, zum Beispiel auf Ihrer/m Veranda, Terrasse, Hof, öffentlichem Hausflur, Garage, Garten, Auffahrt? Wie oft haben Sie ihre Wohnung/Haus verlassen und sind rund um das Haus unterwegs? Nutzen Sie Ihren Rollator/Rollstuhl? Muss Ihnen dabei jemand helfen?*

Zusammenfassung Zone 2: *Lassen Sie mich das zusammenfassen: Sie haben Ihre Wohnung jeden Tag genau ein Mal verlassen, um zum Briefkasten zu gehen und die Zeitung zu holen und dabei ihren Gehstock benutzt? Ist das richtig?*

Zone 3: *Waren Sie in der vergangenen Woche auch in Ihrer Nachbarschaft außerhalb Ihres Wohnhauses, Hofs oder Gartens unterwegs? Waren Sie zum Beispiel Nachbarn besuchen, beim Bäcker oder haben einen Spaziergang in Ihrer Straße gemacht? Wie oft waren Sie in ihrer Nachbarschaft unterwegs? Ich habe auf dem Hinweg einen Supermarkt/Park gesehen, waren Sie dort mal in der vergangenen Woche? Als Sie dorthin gegangen sind, waren Sie mit dem Auto oder zu Fuß unterwegs? Haben Sie jemanden gebraucht, der Ihnen geholfen hat?*

Zusammenfassung Zone 3: *Sie gehen also ein Mal in der Woche zur Tagespflege in der Nachbarschaft und haben wie jeden Dienstag einen Spaziergang mit ihrem Bekannten in der Nachbarschaft hier gemacht. Dann waren Sie zwei Mal beim Bäcker, und ein Mal mit ihrer Tochter einkaufen mit dem Auto. Insgesamt waren Sie also fünf Mal letzte Woche in Ihrer Nachbarschaft unterwegs, stimmt das so? Sie können diese Aktivitäten ohne Hilfspersonen durchführen, brauchen aber Ihren Rollator?*

Zone 4: *Waren Sie in der vergangenen Woche außerhalb Ihrer Nachbarschaft, aber noch innerhalb Ihres Wohnortes/Name der Stadt unterwegs? Waren Sie letzte Woche bei Ihrem Arzt, Einkaufszentrum, Sportgruppe, Tagespflege? Wie oft waren Sie da? Gab es letzte Woche ein besonderes Ereignis, wie ein Fest, eine Familienfeier, oder ein Besuch von Freunden? Gehen Sie dort zu Fuß/selbst hin, oder ist es zu weit weg? Haben Sie ein Fahrrad/Auto/Bus/Taxi genutzt? Nutzen Sie Hilfsmittel wie den Gehstock/Rollator? Gibt es auch eine Person, die Ihnen hilft?*

Zusammenfassung Zone 4: *Lassen Sie mich das wiederholen, Sie waren also letzte Woche zwei Mal außerhalb Ihrer Nachbarschaft unterwegs, ein Mal waren Sie mit dem Taxi Freunde besuchen und ein Mal waren Sie mit Ihrer Tochter mit dem Auto einkaufen, Sie haben Ihren Gehstock und die Hilfe einer Person beim Ein-und Aussteigen in das Auto benötigt? Habe ich das richtig verstanden?*

Zone 5: *Haben Sie in der vergangenen Woche ihre Stadt verlassen? Waren Sie zum Beispiel in (Name der Nachbarstadt/nächstgrößeren Stadt)? Wie oft waren Sie dort? Wie sind Sie dorthin gekommen, selbstständig oder mit Hilfe?*

Zusammenfassung Zone 5: *Sie waren also letzte Woche nicht außerhalb von Ihrer Heimatstadt (Name) unterwegs? Ist das richtig?*

**Bewertung**

Zunächst werden die Ergebnisse auf der Zoneneben multipliziert: Zone (1-5) x (Häufigkeit 1-3) x Selbstständigkeit (1, 1,5 oder 2). Bei Selbstständigkeit wird die selbstständigste Variante bewertet, hat ein Befragter in einer bestimmten Zone beispielsweise ein Mal einen Rollator zum Fortbewegen benutzt und war einmal selbstständig ohne Hilfsmittel unterwegs, wird dies als “selbstständig” (=2) bewertet. Anschließen werden die Ergebnisse für jede Zone addiert.

**Ungültige Aussagen oder Ausschluss von Ergebnissen:**

Bei ganz offensichtlich falschen Angaben oder nicht plausiblen Aussagen sollten die Ergebnisse nicht berücksichtigt werden. Es sollten jedoch Versuche unternommen werden, realistische Angaben zu erhalten (Hinweise zur Mobilität während des Assessments geben, gezielte Fragen zu Hilfsmittel stellen).

Beispiele:

- Der Befragte berichtet, ohne Hilfsmittel außerhalb der Wohnung unterwegs zu sein, ist aber offensichtlich auf Hilfsmittel (sitzt beispielsweise im Rollstuhl und kann nicht selbstständig aufstehen).
- Der Befragte behauptet sein Bett nicht verlassen zu haben, hat jedoch selbstständig die Tür geöffnet.
- Eine Pflegekraft oder Angehörige bestätigen im Anschluss, dass die Angaben nicht korrekt sind.

**Informationen sind erhältlich bei:**

Prof. Dr. Klaus Hauer

Geriatrisches Zentrum an der Universität Heidelberg

Telefon: +00496221 / 319-1783

Email: Klaus.hauer@agaplesion.de

Rohrbacher Str. 149,

69126 Heidelberg, Germany

**References:**

Baker, P. S., Bodner, E. V., & Allman, R. M. (2003). Measuring life-space mobility in community-dwelling older adults. *J Am Geriatr Soc, 51*(11), 1610-1614. doi:10.1046/j.1532-5415.2003.51512.x

Baranowski, T. (1988). Validity and reliability of self reports measures of physical activity: An information processing perspective. Research Quarterly for Exercise and Sport, 59(4), 314–327. doi: 10.1080/02701367.1988.10609379

Bhandari, A., Wagner, T. (2006). Self-reported utilization of health care services: improving measurement and accuracy. Med Care Res Rev, 63(2), 217-235. doi:10.1177/1077558705285298

Durante, R., Ainsworth, B.E. (1996). The recall of physical activity: Using a cognitive model of the question-answering process. Medicine and Science in Sports and Exercise, 28(10), 1282-1291. doi: 10.1097/00005768-199610000-00012

Hauer, K., Lord, S. R., Lindemann, U., Lamb, S. E., Aminian, K., & Schwenk, M. (2011). Assessment of physical activity in older people with and without cognitive impairment. *J Aging Phys Act, 19*(4), 347-372. doi:10.1123/japa.19.4.347

Hauer, K., Yardley, L., Beyer, N., Kempen, G., Dias, N., Campbell, M., Becker, C., Todd, C. (2010). Validation of the Falls Efficacy Scale and Falls Efficacy Scale International in geriatric patients with and without cognitive impairment: results of self-report and interview-based questionnaires. Gerontology.;56(2):190-9. doi: 10.1159/000236027.

Knopman, D.S., Petersen, R.C (2014). Mild cognitive impairment and mild dementia: a clinical perspective. Mayo Clin Proc. Oct;89(10):1452-9. doi: 10.1016/j.mayocp.2014.06.019.

Sallis, J. F., & Saelens, B. E. (2000). Assessment of physical activity by self-report: status, limitations, and future directions. *Res Q Exerc Sport, 71*(2 Suppl), S1-14. doi:10.1080/02701367.2000.11082780

Shephard, R. J. (2003). Limits to the measurement of habitual physical activity by questionnaires. *Br J Sports Med, 37*(3), 197-206; discussion 206. doi:10.1136/bjsm.37.3.197

**Supplemental Document 6: LSA-IS Interview-Version Assessment Form (in German language)**

**Life-Space Assessment in Institutionalized Settings (LSA-IS)**

| Name: | | | Datum: | | |
| --- | --- | --- | --- | --- | --- |
| **Diese Fragen beziehen sich auf Ihre Aktivitäten innerhalb des gestrigen Tages.** | | | | | |
| **LEBENSRAUM-STUFE** | | **HÄUFIGKEIT** | | **Selbstständigkeit** | **PUNKTE** |
| **Waren Sie während des gestrigen Tages…** | | **Wie oft?** | | **Haben Sie Hilfsmittel verwendet?**  **Haben Sie die Hilfe einer anderen Person benötigt?** | Stufe  ×  Häufigkeit  ×  Selbstständigkeit |
| *Stufe 1…*  **innerhalb Ihres Zimmers unterwegs?** | 1 = Ja  0 = Nein | 1 = 1 × pro Tag  2 = 2-3 × pro Tag  3 = >3 × pro Tag | | 1.5 = persönliche Hilfe  1.5 = nur Hilfsmittel  2.5 = ohne Hilfsmittel oder persönliche Hilfe | _________________  *Stufe 1 Punkte* |
| ***Punkte*** | _____ × | _____ × | | _____ = |  |
| *Stufe 2…*  **außerhalb Ihres Zimmers, aber innerhalb Ihrer Station unterwegs?** | 2 = Ja  0 = Nein | 1 = 1 × pro Tag  2 = 2-3 × pro Tag  3 = >3 × pro Tag | | 1.5 = persönliche Hilfe  1.5 = nur Hilfsmittel  2.5 = ohne Hilfsmittel oder persönliche Hilfe | _________________  *Stufe 2 Punkte* |
| ***Punkte*** | _____ × | _____ × | | _____ = |  |
| *Stufe 3…*  **außerhalb Ihrer Station, aber innerhalb der Einrichtung unterwegs (z.B. andere Station, Cafeteria, Kapelle)?** | 3 = Ja  0 = Nein | 1 = 1 × pro Tag  2 = 2-3 × pro Tag  3 = >3 × pro Tag | | 1.5 = persönliche Hilfe  1.5 = nur Hilfsmittel  2.5 = ohne Hilfsmittel oder persönliche Hilfe | ________________  *Stufe 3 Punkte* |
| ***Punkte*** | _____ × | _____ × | | _____ = |  |
| *Stufe 4…*  **außerhalb der Einrichtung, aber innerhalb des dazugehörigen Geländes**  **(z.B. Terrasse, Park)?** | 4 = Ja  0 = Nein | 1 = 1 × pro Tag  2 = 2-3 × pro Tag  3 = >3 × pro Tag | | 1.5 = persönliche Hilfe  1.5 = nur Hilfsmittel  2.5 = ohne Hilfsmittel oder persönliche Hilfe | _________________  *Stufe 4 Punkte* |
| ***Punkte*** | _____ × | _____ × | | _____ = |  |
| *Stufe 5…*  **außerhalb des zur Einrichtung dazugehörigen Geländes?** | 5 = Ja  0 = Nein | 1 = 1 × pro Tag  2 = 2-3 × pro Tag  3 = >3 × pro Tag | | 1.5 = persönliche Hilfe  1.5 = nur Hilfsmittel  2.5 = ohne Hilfsmittel oder persönliche Hilfe | _________________  *Stufe 5 Punkte* |
| ***Punkte*** | _____ × | _____ × | | _____ = |  |
| **Gesamtpunktzahl**  **(LSA-IS-T)** | | | | | _________________  *Summe der Stufen* |
| Max. Stufe (LSA-IS-M) | | | | | ___________ |
| Max. Stufe ohne Hilfemittel und ohne persönliche Hilfe (LSA-IS-I) | | | | | ___________ |
| Max. Stufe nur mit Hilfsmittel, aber ohne persönliche Hilfe (LSA-IS-E) | | | | | ___________ |
| Max. Stufe mit persönlicher Hilfe (LSA-IS-P) | | | | | ___________ |

**Supplemental Document 7: LSA-IS Assessment Manual (in German language)**

**Life-Space Assessment für Personen in Institutionen**

**(LSA-IS)**

**–**

**Manual für Nutzer für die interviewbasierte Version**

**Einführung**

Das “Life-Space Assessment für Personen in Institutionen” (LSA-IS) gibt es als interviewbasierte und als fremdberichtete (Angehörige, Pflegekräfte) Version. Dieses ist das Manual für die interviewbasierte Version. Das LSA-IS wurde auf Basis des “Nursing Home Life-Space Diameters (Tinetti & Ginter 1990) und des “University of Alabama at Birmingham – Life Space Assessment” (UAB-LSA; Baker et al. 2003) entwickelt, um die “Life-Space Mobilität” auch von älteren Menschen, die sich in Einrichtungen wie Krankenhaus, Rehabilitationsklinik oder Pflegeheim befinden, erfassen zu können. Es wurden Anpassungen und Veränderungen im Hinblick auf das Setting und eventuelle Einschränkungen durch fehlendes Erinnerungsvermögen durch akute medizinische Ereignisse, kognitive Einschränkungen, Delir/Narkosefolgen, oder die Ortswechsel bei diesen Personen vorgenommen. Unter anderem aus diesem Grund wurde die Observationsdauer von zwei bzw. vier Wochen auf einen Tag reduziert und eine spezielle Interviewtechnik für die Version des Selbstberichts eingeführt, die eine verzerrte Erfassung der Mobilität durch fehlendes Erinnerungsvermögen verhindern soll. Diese wurde auch schon erfolgreich in früheren Projekten (einem Fragebogen zur Erfassung der körperlichen Aktivität bei Personen mit leichter bis moderater kognitiver Einschränkung) eingesetzt (Hauer et al. 2011).

Das LSA-IS erfasst die Mobilität innerhalb von klar definierten Bereichen (eigener Raum, Station, Einrichtung, und darüber hinausgehende Bereiche), die typisch für eine Einrichtung wie Krankenhaus, Rehabilitationsklinik oder Pflegeheim sind, und erfasst zusätzlich die Häufigkeit dieser Bewegungen und den Bedarf an Hilfsmittel oder Hilfspersonen.

**Zielpopulation**

Die Zielpopulation umfasst ältere Menschen, die sich in einer Institution wie Krankenhaus, Rehabilitationsklinik oder Pflegeheim befinden. Diese Personen weisen häufig Multimorbidität, akute medizinische Probleme, kognitive Einschränkungen, oder eine eingeschränkte Orientierung auf.

**Vorbereitungen für die Durchführung des LSA-CI**

Für Personen in Gesundheitsberufen ist kein spezielles Training erforderlich um den LSA-CI durchzuführen. Benötigt werden lediglich der Fragebogen und ein Stift.

**Vorgehen bei der Durchführung**

Der LSA-CI kann als Interview (oder als Bericht durch Angehörige oder Pflegekräfte siehe LSA-IS proxy-version und Manual) durchgeführt wurden und berücksichtigt die speziellen Bedürfnisse von älteren, multi-morbiden Personen mit möglicher körperlicher oder kognitiver Einschränkung. Bei der interview-basierten Variante sollte die Erhebung alleine stattfinden, um die Aufmerksamkeit der zu befragenden Person nicht zu beeinträchtigen. Sollten Zimmernachbarn, Angehörige, oder Pflegekräfte anwesend sein, dürfen diese nicht das Antworten übernehmen oder sich einmischen. Im Anschluss an die Durchführung können die Angehörigen oder Pflegekräfte jedoch die Aussagen bestätigen oder falsifizieren.

Das Befragung sollte in einem ruhigen Umfeld durchgeführt werden, am besten im Zimmer des Befragten, so kann sich der Interviewer sich auf die aktuelle Situation beziehen und die Fragen konkret stellen. Das Wissen um die aktuelle Situation ist auch hilfreich, um die Genauigkeit der Antworten direkt erfassen zu können und gegebenenfalls Rückfragen zu stellen (z.B. steile Treppe am Eingangsbereich, Stellplatz für Hilfsmittel etc.). Es sollte gezielt nach offensichtlichen Einschränkungen in der Beweglichkeit (z.B. Schwierigkeiten beim Aufstehen, Treppengehen etc.) und nach Hilfsmittel (z.B. sichtbarer Gehstock, Rollator etc.) gefragt werden, um einen realistischen Eindruck der aktuellen Fähigkeiten und Tätigkeiten zu erlangen.

***Dermenzspezifische Interview-Technik:***

Die Befragung sollte im persönlichen Gespräch und interviewbasiert erfolgen, um Ungenauigkeiten auszuschließen bzw. zu reduzieren, die Vollständigkeit der Antworten abzusichern und gleichzeitig auf eine mögliche Überförderung des Befragten eingehen zu können (Hauer et al. 2010). Diese Form der Erfassung erlaubt umfassende mündliche Erläuterungen und lässt Raum für Rückfragen, was Ungenauigkeiten oder Mehrdeutigkeit verhindert (Durante & Ainsworth 1996) und Versagensängste in Bezug auf Verständnis und Erinnerungsfähigkeit bei den Befragten verhindern (Hauer et al 2011).

**Schritt 1:** Die Befragung sollte mit einer kurzen Erläuterung der Ziele und der Dauer der Befragung (3 bis 5 Minuten) starten.

**Schritt 2:** Wichtig ist, den Beginn und das Ende des Beobachtungszeitraums klar zu benennen (den gestrigen Tag). Falls während der Befragungen Unklarheiten auftreten, kann es angemessen sein, die Aktivitäten in jeder Zone in Abschnitten abzufragen und den Tag zu segmentieren mit Hilfe von typischen Aktivitäten (nach dem Aufstehen, vor dem Frühstück, bis zum Mittagessen, vor der Therapie/Arztbesuch/Gruppe etc.).

**Schritt 3:** Fragen Sie einzeln nach jeder Life-Space Zone, beginnend mit Zone 1 und dann kontinuierlich weitergehend ohne eine Zone auszulassen.

Spezielle Zeitfenster sowie tägliche Rituale und Gewohnheiten können dabei als “Anker” genutzt werden, um den Beobachtungszeitraum zu segmentieren oder einzuteilen (z.B. Wochenende und Wochentage, Aufstehen, Mahlzeiten, Körperpflege, Gang zu Cafeteria/Shop/Kiosk, Fernsehen, etc.).

Zusätzlich sollte gezielt nach typischen Aktivitäten gefragt werden, sofern diese nicht selbst erwähnt werden (Besuche von Freunden und Verwandten, Arztbesuche, Therapien) um die Vollständigkeit der Erfassung abzusichern. Geschlossene Fragen können dabei besser geeignet sein, um eine kognitive Überforderung zu vermeiden (“Waren sie einkaufen” statt “Was haben Sie sonst gemacht?”).

Im Fokus der Erfassung steht die aktuelle Mobilität, also das, was die Befragten tatsächlich am vorigen Tag gemacht haben, und nicht das, wozu sie in der Lage gewesen wären.

Ein Anhaltspunkt für die Genauigkeit der Erfassung der einzelnen Zonen ist, dass es nicht möglich ist, eine Zone zu erreichen, ohne sich in den niedrigeren Zonen bewegt zu haben (z.B. ist es nicht möglich, in außerhalb der Institution unterwegs gewesen zu sein, ohne die Institution durchquert zu haben).

**Schritt 4:** Die Befragung schließt mit einer Zusammenfassung des Gesagten für jede jeweilige Zone einschließlich der Häufigkeit und des Bedarfs an Hilfe, so haben die Befragten Zeit, ihre eigenen Aussagen zu prüfen und zu bestätigen oder korrigieren.

**Beispiele für die Befragung und die Zusammenfassung:**

Zone 1: *Haben Sie sich gestern außerhalb Ihres Zimmers aufgehalten? Haben Sie nach dem Aufstehen ihr Bett verlassen? Haben Sie sich gestern krank gefühlt und den Tag im Bett verbracht? Haben Sie Hilfe benötigt, als Sie Ihr Bett verlassen, halten Sie sich an Ihrem Rollator/ Nachttisch etc. fest? Wenn Sie in das Bad/zum Fernseher/Schrank gehen, nutzen Sie dann den Rollator/Gehstock?*

Zusammenfassung Zone 1: *Also, Sie haben gestern ihr Bett verlassen und sich mindestens zehn Mal in Ihrem Raum bewegt und dabei keine Hilfsmittel benötigt? Ist das so korrekt?*

Zone 2: *Waren Sie gestern außerhalb Ihres Zimmers und auf Ihrer Station unterwegs? Wie oft haben Sie Ihr Zimmer verlassen und sind im Flur oder in der Teeküche auf der Station unterwegs? Nutzen Sie Ihren Rollator/Rollstuhl? Muss Ihnen dabei jemand helfen?*

Zusammenfassung Zone 2: *Lassen Sie mich das zusammenfassen: Sie haben gestern ihr Zimmer 4 Mal verlassen, 3 Mal zu den Mahlzeiten im Gemeinschaftsraum und ein Mal, um zur Teeküche zu gehen? Dabei haben Sie Ihren Rollator benutzt? Ist das richtig?*

Zone 3: *Waren Sie gestern auch außerhalb Ihrer Station unterwegs? Waren Sie zum Beispiel eine andere Station besuchen, in der Cafeteria oder haben Sie den Therapieraum/Gruppenraum genutzt? Wie oft waren Sie außerhalb Ihrer Station unterwegs? Kennen Sie die Cafeteria / Kapelle / Kiosk, waren Sie gestern dort? Als Sie dorthin gegangen sind, waren Sie mit alleine unterwegs? Haben Sie hierbei ein Hilfsmittel oder die Hilfe einer Person benötigt?*

Zusammenfassung Zone 3: *Sie waren gestern also zwei Mal außerhalb der Station, ein Mal vormittags in der Cafeteria im Erdgeschoss, und noch ein Mal am Nachmittag um in den Park zu gelangen? Sie konnten diese Aktivitäten ohne Hilfspersonen durchführen, brauchten aber Ihren Rollator?*

Zone 4: *Waren Sie gestern außerhalb des Krankenhauses/Pflegeheims (Name)aber auf dem Krankenhaus/Heimgelände unterwegs? Wie oft waren Sie da? Gehen Sie dort zu Fuß/selbst hin, oder ist es zu weit weg? Nutzen Sie Hilfsmittel wie den Gehstock/Rollator? Gibt es auch eine Person, die Ihnen hilft?*

Zusammenfassung Zone 4: *Lassen Sie mich das wiederholen, Sie waren gestern also zwei Mal außerhalb Ihrer Pflegeheims unterwegs, und zwar waren Sie ein Mal beim Arzt, und ein Mal haben Sie sich in den Park gesetzt. Sie haben für den Weg in den Park Ihren Rollator benötigt und nur für die Taxifahrt die Hilfe einer Person beim Ein-und Aussteigen in das Auto? Habe ich das richtig verstanden?*

Zone 5: *Haben Sie gestern die Einrichtung verlassen und waren in der Stadt unterwegs? Wie oft waren Sie dort? Gab es gestern ein besonderes Ereignis, wie einen Arztbesuch, oder einen Ausflug? Wie sind Sie dorthin gekommen, selbstständig oder mit Hilfe?*

Zusammenfassung Zone 5: *Sie waren also gestern nicht außerhalb des Krankenhausgeländes unterwegs? Ist das richtig?*

**Bewertung**

Zunächst werden die Ergebnisse auf der Zoneneben multipliziert: Zone (1-5) x (Häufigkeit 1-3) x Selbstständigkeit (1, 1,5 oder 2). Bei Selbstständigkeit wird die selbstständigste Variante bewertet, hat ein Befragter in einer bestimmten Zone beispielsweise ein Mal einen Rollator zum Fortbewegen benutzt und war einmal selbstständig ohne Hilfsmittel unterwegs, wird dies als “selbstständig” (=2) bewertet. Anschließen werden die Ergebnisse für jede Zone addiert.

**Ungültige Aussagen oder Ausschluss von Ergebnissen:**

Bei ganz offensichtlich falschen Angaben oder nicht plausiblen Aussagen sollten die Ergebnisse nicht berücksichtigt werden. Es sollten jedoch Versuche unternommen werden, um realistische Angaben zu bekommen (Hinweise auf Mobilität während Assessment, gezielte Fragen zu Hilfsmittel).

Beispiele:

- Der Befragte berichtet, ohne Hilfsmittel außerhalb der Einrichtung unterwegs zu sein, ist aber offensichtlich auf Hilfsmittel angewiesen (sitzt beispielsweise im Rollstuhl und kann nicht selbstständig aufstehen).
- Der Befragte behauptet sein Bett nicht verlassen zu haben, sitzt jedoch während der Befragung am Tisch.
- Eine Pflegekraft oder Angehörige bestätigen im Anschluss, dass die Angaben nicht korrekt sind.

**Informationen sind erhältlich bei:**

Prof. Dr. Klaus Hauer

Geriatrisches Zentrum an der Universität Heidelberg

Telefon: +00496221 / 319-1783

Email: Klaus.hauer@agaplesion.de

Rohrbacher Str. 149,

69126 Heidelberg, Germany

**References:**

Baker, P. S., Bodner, E. V., & Allman, R. M. (2003). Measuring life-space mobility in community-dwelling older adults. *J Am Geriatr Soc, 51*(11), 1610-1614. doi:10.1046/j.1532-5415.2003.51512.x

Durante, R., Ainsworth, B.E. (1996). The recall of physical activity: Using a cognitive model of the question-answering process. Medicine and Science in Sports and Exercise, 28(10), 1282-1291. doi: 10.1097/00005768-199610000-00012

Hauer, K., Lord, S. R., Lindemann, U., Lamb, S. E., Aminian, K., & Schwenk, M. (2011). Assessment of physical activity in older people with and without cognitive impairment. *J Aging Phys Act, 19*(4), 347-372. doi:10.1123/japa.19.4.347

Hauer, K., Yardley, L., Beyer, N., Kempen, G., Dias, N., Campbell, M., Becker, C., Todd, C. (2010). Validation of the Falls Efficacy Scale and Falls Efficacy Scale International in geriatric patients with and without cognitive impairment: results of self-report and interview-based questionnaires. Gerontology.;56(2):190-9. doi: 10.1159/000236027.

Tinetti, M. E., & Ginter, S. F. (1990). The nursing home life-space diameter. A measure of extent and frequency of mobility among nursing home residents. J Am Geriatr Soc, 38(12), 1311-1315**.**

**Supplemental Document 8: LSA-IS Proxy Report Assessment Form (in German language)**

**Life-Space Assessment in Institutionalized Settings (LSA-IS)**

| Name: | | | Datum: | | |
| --- | --- | --- | --- | --- | --- |
| **Diese Fragen beziehen sich auf die Aktivitäten innerhalb des gestrigen Tages.** | | | | | |
| **LEBENSRAUM-STUFE** | | **HÄUFIGKEIT** | | **Selbstständigkeit** | **PUNKTE** |
| **War der Patient/Bewohner/Gast während des gestrigen Tages…** | | **Wie oft?** | | **Hat er/sie Hilfsmittel verwendet?**  **Hat er/sie die Hilfe einer anderen Person benötigt?** | Stufe  ×  Häufigkeit  ×  Selbstständigkeit |
| *Stufe 1…*  **innerhalb des eigenen Zimmers unterwegs?** | 1 = Ja  0 = Nein | 1 = 1 × pro Tag  2 = 2-3 × pro Tag  3 = >3 × pro Tag | | 1.5 = persönliche Hilfe  1.5 = nur Hilfsmittel  2.5 = ohne Hilfsmittel oder persönliche Hilfe | _________________  *Stufe 1 Punkte* |
| ***Punkte*** | _____ × | _____ × | | _____ = |  |
| *Stufe 2…*  **außerhalb desZimmers, aber innerhalb der Station unterwegs?** | 2 = Ja  0 = Nein | 1 = 1 × pro Tag  2 = 2-3 × pro Tag  3 = >3 × pro Tag | | 1.5 = persönliche Hilfe  1.5 = nur Hilfsmittel  2.5 = ohne Hilfsmittel oder persönliche Hilfe | _________________  *Stufe 2 Punkte* |
| ***Punkte*** | _____ × | _____ × | | _____ = |  |
| *Stufe 3…*  **außerhalb der Station, aber innerhalb der Einrichtung unterwegs (z.B. andere Station, Cafeteria, Kapelle)?** | 3 = Ja  0 = Nein | 1 = 1 × pro Tag  2 = 2-3 × pro Tag  3 = >3 × pro Tag | | 1.5 = persönliche Hilfe  1.5 = nur Hilfsmittel  2.5 = ohne Hilfsmittel oder persönliche Hilfe | ________________  *Stufe 3 Punkte* |
| ***Punkte*** | _____ × | _____ × | | _____ = |  |
| *Stufe 4…*  **außerhalb der Einrichtung, aber innerhalb des dazugehörigen Geländes**  **(z.B. Terrasse, Park)?** | 4 = Ja  0 = Nein | 1 = 1 × pro Tag  2 = 2-3 × pro Tag  3 = >3 × pro Tag | | 1.5 = persönliche Hilfe  1.5 = nur Hilfsmittel  2.5 = ohne Hilfsmittel oder persönliche Hilfe | _________________  *Stufe 4 Punkte* |
| ***Punkte*** | _____ × | _____ × | | _____ = |  |
| *Stufe 5…*  **außerhalb des zur Einrichtung dazugehörigen Geländes?** | 5 = Ja  0 = Nein | 1 = 1 × pro Tag  2 = 2-3 × pro Tag  3 = >3 × pro Tag | | 1.5 = persönliche Hilfe  1.5 = nur Hilfsmittel  2.5 = ohne Hilfsmittel oder persönliche Hilfe | _________________  *Stufe 5 Punkte* |
| ***Punkte*** | _____ × | _____ × | | _____ = |  |
| **Gesamtpunktzahl**  **(LSA-IS-T)** | | | | | _________________  *Summe der Stufen* |
| Max. Stufe (LSA-IS-M) | | | | | ___________ |
| Max. Stufe ohne Hilfemittel und ohne persönliche Hilfe (LSA-IS-I) | | | | | ___________ |
| Max. Stufe nur mit Hilfsmittel, aber ohne persönliche Hilfe (LSA-IS-E) | | | | | ___________ |
| Max. Stufe mit persönlicher Hilfe (LSA-IS-P) | | | | | ___________ |

**Supplemental document 9: LSA-IS Proxy Report Assessment Manual (in German language)**

**Life-Space Assessment für Personen in Institutionen**

**(LSA-IS)**

**–**

**Manual für Nutzer für die proxy-Version (Erfassung durch Angehörige oder Pflegekräfte)**

**Einführung**

Das “Life-Space Assessment für Personen in Institutionen” (LSA-IS) gibt es als interviewbasierte und als proxy-report Version, hierzu dokumentieren Angehörige oder Pflegekräfte die Mobilität des zu Beobachtenden innerhalb des gestrigen Tages. Das LSA-IS wurde auf Basis des “Nursing Home Life-Space Diameters (Tinetti & Ginter 1990) und des “University of Alabama at Birmingham – Life Space Assessment” (UAB-LSA; Baker et al. 2003) entwickelt, um die “Life-Space Mobilität” auch von älteren Menschen, die sich in Einrichtungen wie Krankenhaus, Rehabilitationsklinik oder Pflegeheim befinden, erfassen zu können. Es wurden Anpassungen und Veränderungen im Hinblick auf das Setting vorgenommen. Aus verschiedenen Gründen wurde die Observationsdauer von zwei bzw. vier Wochen auf einen Tag reduziert.

Das LSA-IS erfasst die Mobilität innerhalb von klar definierten Bereichen (eigener Raum, Station, Einrichtung, und darüber hinausgehende Bereiche), die typisch für eine Einrichtung wie Krankenhaus, Rehabilitationsklinik oder Pflegeheim sind, und erfasst zusätzlich die Häufigkeit dieser Bewegungen und den Bedarf an Hilfsmittel oder Hilfspersonen.

**Zielpopulation**

Die Zielpopulation umfasst ältere Menschen, die sich in einer Institution wie Krankenhaus, Rehabilitationsklinik oder Pflegeheim befinden. Diese Personen weisen häufig Multimorbidität, akute medizinische Probleme, kognitive Einschränkungen, oder eine eingeschränkte Orientierung auf.

**Vorbereitungen für die Durchführung des LSA-CI**

Für Personen in Gesundheitsberufen ist kein spezielles Training erforderlich um den LSA-CI durchzuführen. Benötigt werden lediglich der Fragebogen und ein Stift.

**Vorgehen bei der Durchführung**

Der LSA-CI sollte nur von einer Person durchgeführt hat, die Einblick in das aktuelle Bewegungsverhalten des zu Beobachtenden hat (Bedarf an Hilfsmittel oder Hilfspersonen, Gewohnheiten), die Abläufe in der Einrichtung (Therapien, Gruppenangebote, Mahlzeiten) und die die räumliche Umgebung (Garten- oder Parkanlagen, Einkaufsmöglichkeiten in der Umgebung) kennt. Der Beobachter sollte die Gelegenheit haben, den zu Beobachtenden mehrfach am Tag zu sehen, ohne dabei in dessen Mobilitätsverhalten einzugreifen.

Im Fokus der Erfassung steht die aktuelle Mobilität, also das, was die Befragten tatsächlich am vorigen Tag gemacht haben, und nicht das, wozu sie in der Lage gewesen wären.

Ein Anhaltspunkt für die Genauigkeit der Erfassung der einzelnen Zonen ist, dass es nicht möglich ist, eine Zone zu erreichen, ohne sich in den niedrigeren Zonen bewegt zu haben (z.B. ist es nicht möglich, in außerhalb der Institution unterwegs gewesen zu sein, ohne die Institution durchquert zu haben).

**Bewertung**

Zunächst werden die Ergebnisse auf der Zoneneben multipliziert: Zone (1-5) x (Häufigkeit 1-3) x Selbstständigkeit (1, 1,5 oder 2). Bei Selbstständigkeit wird die selbstständigste Variante bewertet, hat ein Befragter in einer bestimmten Zone beispielsweise ein Mal einen Rollator zum Fortbewegen benutzt und war einmal selbstständig ohne Hilfsmittel unterwegs, wird dies als “selbstständig” (=2) bewertet. Anschließen werden die Ergebnisse für jede Zone addiert.

**Informationen sind erhältlich bei:**

Prof. Dr. Klaus Hauer

Geriatrisches Zentrum an der Universität Heidelberg

Telefon: +00496221 / 319-1783

Email: Klaus.hauer@agaplesion.de

Rohrbacher Str. 149,

69126 Heidelberg, Germany

**References:**

Baker, P. S., Bodner, E. V., & Allman, R. M. (2003). Measuring life-space mobility in community-dwelling older adults. *J Am Geriatr Soc, 51*(11), 1610-1614. doi:10.1046/j.1532-5415.2003.51512.x

Tinetti, M. E., & Ginter, S. F. (1990). The nursing home life-space diameter. A measure of extent and frequency of mobility among nursing home residents. J Am Geriatr Soc, 38(12), 1311-1315**.**
